# Arrest in the Progression of Type 1 Diabetes at the Mid-Stage of Insulitic Autoimmunity Using an Autoantigen-Decorated All-*trans* Retinoic Acid and Transforming Growth Factor Beta-1 Single Microparticle Formulation

**DOI:** 10.3389/fimmu.2021.586220

**Published:** 2021-03-08

**Authors:** Brett E. Phillips, Yesica Garciafigueroa, Carl Engman, Wen Liu, Yiwei Wang, Robert J. Lakomy, Wilson S. Meng, Massimo Trucco, Nick Giannoukakis

**Affiliations:** ^1^Institute of Cellular Therapeutics, Allegheny Health Network, Pittsburgh, PA, United States; ^2^Graduate School of Pharmaceutical Sciences, Duquesne University, Pittsburgh, PA, United States; ^3^McGowan Institute for Regenerative Medicine, University of Pittsburgh, Pittsburgh, PA, United States

**Keywords:** type 1 diabetes, vaccine, tolerance, microparticles, immunoregulation, autoimmunity

## Abstract

Type 1 diabetes (T1D) is a disorder of impaired glucoregulation due to lymphocyte-driven pancreatic autoimmunity. Mobilizing dendritic cells (DC) *in vivo* to acquire tolerogenic activity is an attractive therapeutic approach as it results in multiple and overlapping immunosuppressive mechanisms. Delivery of agents that can achieve this, in the form of micro/nanoparticles, has successfully prevented a number of autoimmune conditions *in vivo*. Most of these formulations, however, do not establish multiple layers of immunoregulation. all-*trans* retinoic acid (RA) together with transforming growth factor beta 1 (TGFβ1), in contrast, has been shown to promote such mechanisms. When delivered in separate nanoparticle vehicles, they successfully prevent the progression of early-onset T1D autoimmunity *in vivo*. Herein, we show that the approach can be simplified into a single microparticle formulation of RA + TGFβ1 with surface decoration with the T1D-relevant insulin autoantigen. We show that the onset of hyperglycemia is prevented when administered into non-obese diabetic mice that are at the mid-stage of active islet-selective autoimmunity. Unexpectedly, the preventive effects do not seem to be mediated by increased numbers of regulatory T-lymphocytes inside the pancreatic lymph nodes, at least following acute administration of microparticles. Instead, we observed a mild increase in the frequency of regulatory B-lymphocytes inside the mesenteric lymph nodes. These data suggest additional and potentially-novel mechanisms that RA and TGFβ1 could be modulating to prevent progression of mid-stage autoimmunity to overt T1D. Our data further strengthen the rationale to develop RA+TGFβ1-based micro/nanoparticle “vaccines” as possible treatments of pre-symptomatic and new-onset T1D autoimmunity.

## Introduction

Type 1 diabetes (T1D) is widely-viewed as a disorder of impaired glucoregulation primarily due to a pancreatic beta (β) cell-selective, largely lymphocyte-driven autoimmunity (1–3). Significant knowledge about pathogenesis of T1D derives from the spontaneously diabetic nonobese diabetic (NOD) mouse strain, believed to share etiopathogenesis with human T1D (4). TH1 and TH17 T-lymphocytes (T-cells) lead this process resulting in β cell destruction (5–8). Dead and dying β cells are acquired by macrophages and DC in steady-state flux through islet structures which migrate into the pancreatic lymph nodes (PLN) where they amplify a vicious circle of T1D autoimmunity by triggering expansion of more β cell-autoreactive T-cells. Regulatory lymphocytes, especially T-cells that stably-express the Foxp3 transcription factor (Foxp3+ Tregs) prevent autoimmune diabetes in the NOD mouse (9) and there is strong evidence that they can regulate the pool of autoreactive effector CD4+ and CD8+ T-cells in humans (10–12). Indeed, autologous Tregs are now in different phases of clinical trials to preserve residual beta cell mass in new onset T1D patients (13–17). While *ex vivo* generation of Tregs has been realized, logistic hurdles stand in the way of expanding personalized Treg cell therapy at a population level (13–17). A simpler way to generate Tregs *in vivo*, and fortify other layers of immune tolerance using a “vaccine,” would be decisive in preventing and treating new onset disease.

We have demonstrated that microsphere formulations of antisense DNA oligonucleotides targeting the primary transcripts of CD40, CD80, and CD86 prevent and reverse T1D (18). We observed an increased prevalence of Foxp3+ Tregs together with a decrease in TH1 cytokine levels in successfully-treated, diabetes-free mice, especially in PLN, when injected into the abdominal region overlying the expected location of the pancreas. The effect was β-cell specific since T-cells from successfully-treated mice proliferated vigorously to alloantigen, but not to β-cell antigens *in vitro*. Protection offered by the microspheres was adoptively-transferable to immunodeficient recipients even in the presence of diabetogenic immune cells (18). In parallel, we had examined the effects of the antisense DNA oligonucleotides on dendritic cells (DC) and we discovered that they stimulated the DC to produce retinoic acid (RA) (19, 20). These RA-producing DC were further shown to increase the frequency of IL-10+ regulatory B-lymphocytes (Bregs) as well as stimulate the proliferation of existing Bregs (19, 20). We surmised that a microsphere formulation of the antisense DNA oligonucleotides could also confer RA-producing ability to DC. In fact, we subsequently discovered that DC isolated from the injection site of microsphere-formulated antisense DNA oligonucleotides acquired the ability to produce retinoic acid (RA) as they took up the microsphere formulations and migrated from this injection site to PLN. These DC, accumulated inside PLN, continued to produce RA. These microspheres were subsequently shown to mobilize DC and Tregs inside the PLN of mice exhibiting an ongoing *ex vivo*-induced inflammation (21).

While a number of factors, and different *in vitro* and *in vivo* environments can support the differentiation of Foxp3+ Tregs from precursor T-cells (22–25), transforming growth factor beta (TGFβ) appears to be a common denominator (26–32). Evidence of the *in vivo* effect of TGFβ in Treg cell pool expansion is described in NOD mice treated with TGFβ. In these mice, TGFβ inhibits T1D development, and increases Treg frequency inside islets. Studies in human T-cells demonstrate TGFβ is necessary to induce Tregs. Treatment of human CD4+ cells with TGFβ increases the number of Tregs and expression of CD25 and intracellular CTLA-4. Expansion is due to increased proliferation and protection of cells from activation-induced apoptosis (33). TGFβ promotes induction of Tregs accompanied by an increase in Foxp3 expression. TGFβ converts CD4+ CD25– Foxp3– non-Tregs into CD4+ CD25+ Foxp3+ Tregs. On its own, however, TGFβ is unable to mediate Treg cell induction. RA appears to be an additional factor that licenses the induction process. In mucosal tissue, mature tolerogenic DC producing RA induce Foxp3+ Tregs via a TGFβ -dependent mechanism. RA enhances TGFβ signaling by increasing expression and phosphorylation of Smad3, and this results in increased Foxp3 expression, even in presence of proinflammatory IL-6 or IL-21 (34). Two studies addressed the role of retinoids in T1D using NOD mice. One examined the effect of high vitamin A concentrations on T1D development (35). RA was protective. The other demonstrated that RA inhibited disease development in multiple low dose streptozotocin and naturally-occurring T1D in NOD mice. Prevention was abrogated in Foxp3+ Treg-depleted mice. T1D hyperglycemia was reversible in new onset NOD mice so long as RA was available (36).

The conceptual feasibility of an RA-based immunoregulatory microparticle method to suppress inflammation has been demonstrated (37, 38). PLGA-based particle RA formulation suppressed IL-17 production and RORγ(t) expression in T-cells polarized toward TH17 phenotype *in vitro* with similar potency to that of free drug. RA nanoparticles enhanced TGFβ -dependent Foxp3 expression and IL-10 production in T-cells *in vitro* with similar potency to free RA. T-cells polarized toward TH17 phenotype in presence of free and nanoparticulate RA similarly suppress ability to induce IL-6 production by fibroblasts. DC isolated from cervical lymph nodes and pulsed with PLGA nanoparticles efficiently induces proliferation of Foxp3+ Tregs *in vitro*. Further data demonstrate that nanoparticle formulations that contain *either RA or* TGFβ in combination with another drug (39, 40), or in one instance administered together as *separate* particles (40) are effective in suppression of inflammation through regulatory cell networks.

Until now, no formulation that combined RA and TGFβ together with a T1D-relevant autoantigen, to induce antigen-specific immune hyporesponsiveness, was considered as a potential therapeutic vehicle. We present evidence, herein, that a novel and stable formulation of a RA and TGFβ-formulated single microparticle, decorated with a T1D-relevant autoantigen (Insulin B9-23 peptide) (41–44) can prevent the onset of hyperglycemia when administered into NOD mice that are at the mid-stage of active islet-selective autoimmunity. Acute treatment of late stage autoimmune pre-diabetic NOD mice with the combined RA/TGFβ/T1D-relevant autoantigen microparticle formulation resulted in a mild increase in the frequency of regulatory B-lymphocytes (Bregs) inside the mesenteric lymph nodes (MLN), but not the PLN. These data suggest additional and potentially-novel mechanisms that RA and TGFβ could be modulating in the prevention of progression of mid-stage autoimmunity to hyperglycemic T1D.

## Materials and Methods

### Experimental Animals

Female NOD/LtJ mice were purchased from the Jackson Laboratories (Bar Harbor, ME) at 6–8 weeks of age and housed up to 34 weeks. Prior to randomization into the treatment arms, NOD female mice between 9 and 11 weeks of age were pre-screened to insure absence of overt hyperglycemia. Blood glucose was assessed using the One Touch Ultra Blood Glucose Meter (Lifescan, Malvern, PA). Animals were maintained in a specific pathogen-free environment in the Animal Facility of the Allegheny Health Network Research Institute. All procedures utilized were in full compliance with and approved by the Institutional Animal Care and Use Research Committee of the Allegheny Health Network Research Institute.

### Synthesis and Characterization of RA and TGFβ-Formulated Microparticles

We have previously described surface-functionalized poly lactic-co-glycolic acid (PLGA) particles as drug carriers (45–49). In particular, surface nickel (Ni)-formulated PLGA microparticles (PLGA-Ni) serves as the backbone of our formulations. PLGA-Ni particles were prepared by incorporating the metal chelating lipid 18:1 DOGS-NTA-Ni into the PLGA matrix using a double-emulsion solvent evaporation method. Briefly, 90 mg of PLGA (50:50) dissolved in 2.4 ml dichloromethane (DCM) was admixed with 0.6 ml of 10 mg/ml DOGS-NTA-Ni dissolved in the same solvent. The organic mixture was slowly added to 20 ml 1% polyvinyl alcohol aqueous solution and homogenized at 25,000 rpm for 5 min on ice. These microparticles are designated as PLGA-Ni. Additional PLGA-Ni formulations include all-*trans* retinoic acid (RA) (Sigma-Aldrich, St. Louis, MO) or Nile Red dye (Thermo Fisher Scientific, Waltham, MA). 5.0 mg of RA or Nile red dye were loaded in the organic phase (DCM) with PLGA and the lipid. Using a solvent extraction method, loading of RA was determined to be 4.8 μg per mg of dry powder, with the efficiency determined to be 61% by BCA protein assay (data not shown). Microparticles containing Nile Red dye are designated as PLGA-Red in this manuscript. Particles were precipitated by evaporating DCM and collected by centrifugation, before lyophilization in 2% trehalose for long-term storage at 4°C in a desiccator until use. His-tagged proteins were attached after washing and resuspension in low protein binding microcentrifuge tubes (Thermo Fisher Scientific). Five microgram of the following His-tagged proteins were incubated per 10 mg of microparticles for 1 h: G protein (pG), GFP (Thermo Fisher Scientific), TGFβ1 (Prospec, Rehovot, Israel), or human insulin B9-23 peptide (8 μg) (R&D Biosystems, Minneapoplis, MN). Using the BCA assay, 45.5% of the His-tagged TGF β1protein was associated with the microparticles (data not shown). RA-formulated, TGFβ1 and insulin B9-23 peptide-modified microparticles (all three components constituting the microparticle) were designated Ins-RT-NP.

### Microparticle Microscopy and Size Determination

Fluorescence microscopy was performed with a IX53 inverted microscope (Olympus, Shinjuku, Japan) with a 20x objective. His-tagged GFP was admixed with particle suspensions, fixed on slide cover and mounted with Slowfade Diamond solution. Scanning electron microscopy (SEM) of the microparticle formulations was conducted after placing the sample solution (200 μL) directly onto sample stubs. Stubs were then covered in foil and left in a desiccator overnight before testing. Samples were examined using a S-3400N Scanning Electron Microscope 14 (Hitatchi, Tokyo, Japan) equipped with an AXS XFlash Detector 5010 (Bruker, Billerica, MA) and a BrukerNano e-Flash 1000+ (Bruker, Billerica, MA). Hydrodynamic size of the microparticle formulations was measured using a Zetasizer Nano-S; particles were washed to remove trehalose and resuspended in ultrapure water (pH 7.4); measurements were made with dilutions at 5, 2.5, 0.25, 0.0125, 0.00025, and 0.0000025 mg per ml to obtain consensus.

### Formulation Release Kinetics

His-tagged GFP was used as a TGFβ1 surrogate to determine release kinetics *in vitro*. Briefly, 5 mg of the PLGA-Ni microparticles were washed and resuspend in 100 μl of pH 8 Tris buffer with 0.1% BSA and loaded with 200 μl of 0.025 mg/ml His-GFP to 0.2 ml pH 8 Tris buffer (0.5 ml Tris buffer in total) and incubated for 1 h at room temperature. Particles were then washed to remove unbound proteins before re-suspended in 1 ml of release medium (1% BSA) and transferred into a 1 ml syringe fitted with a 0.22 μm syringe. Samples (100 μl) were dispensed from the syringe kept at 37°C at the specific time points. Equal volume of the release medium was replaced in the syringe after each sampling. The concentration of His-GFP collected at each time point was determined by measuring the fluorescence intensity (λ = 508 nm) using a Tecan M1000 spectrophotometer (Tecan, Mannedorf, Switzerland) with an established standard curve. Similarly PLGA-Ni microparticles bound with TGFβ1 were placed in a complete elution buffer. A small sample (5 μl) of the elution was loaded into individual wells of a 6–12% polyacrylamide gradient gel, together with molecular weight marker proteins (Thermo Fisher Scientific) and separated by SDS-PAGE in single dimension. The separated proteins were visualized in the gel *in situ* using silver staining. RA was found to release gradually from the particles at 37°C in PBS. In a preparation of 10 mg of PLGA-Ni RA containing microparticles was dissolved in 1 ml of PBS, and the rate of release was monitored over 18 days *in vitro* by UV absorbance.

### *Ex vivo* Microparticle Uptake

Spleens were harvested from NOD female mice euthanized at 10 weeks of age. A single cells suspension was made by first physically dissociating the spleen followed by a 10 min incubation in RBC lysis buffer (eBioscience) to remove red blood cells. The cell suspension was then strained through a 100 μm filter and plated at 1 × 10^6^ cells/well in complete RPMI cell culture media (Sigma Aldrich) *plus* 10% fetal bovine serum (Gibco). Cells were plated in a 96-well tissue culture plate in a final volume of 200 μl and allowed to settle prior to addition of microparticles. PLGA-Red microparticles were resuspended in tissue culture media. 0.25 mg of reconstituted particles were then added to the cell wells and incubated together at 37°C for 18 h. Cells were then collected and washed three times to remove extra-cellular particles. The uptake of particles by cells was measured by flow cytometry (BD Influx) and the data were analyzed by FlowJo software version 10.7.1.

### *In vivo* Microparticle Administration

Microparticles were prepared at a concentration of 10 mg per 200 μL vehicle volume for administration to mice. PLGA-Ni and PLGA-Red microparticles were resuspended in sterile saline in low protein binding microcentrifuge tubes. PLGA (RA)-Ni were incubated with His-tagged recombinant human TGFβ1 at a concentration of 25 μg/mL, or 5 μg total weight and His-tagged human insulin B9-23 peptide (3-letter amino acid sequence: H-Ser-His-Leu-Val-Glu-Ala-Leu-Tyr-Leu-Val-Cys-Gly-Glu-Arg-Gly-OH) was added at a concentration of 40 μg/mL, or 8 μg total weight. The microparticle suspension was maintained dispersed by pipet mixing and incubated at room temperature for 30 min. After incubation, the suspension was again mixed by pipetting and loaded into 1 ml syringes with 27 gauge needles. Eleven week-old female NODLtJ/Shi mice were administered the microparticle formulations by subcutaneous (s.c.) injection into the abdomen. None of the mice were hyperglycemic or exhibited dysglycemia at the time of microparticle administration.

### Tissue Single Cell Collection and Processing

PLN, MLN and skin (a 1 × 1 cm^2^ patch at the injection site) were resected from randomly-selected diabetes-free mice from all the treatment arms. Lymph nodes were physically dissociated and the tissue was strained through 100 μm filters (Fisher Scientific) to produce single cell suspensions. Viability of cells was assessed by Trypan Blue staining and counted in a Countess II FL device (Thermo Fisher). Skin was isolated and processed into single cells as described previously (50). Briefly, hair around the injection site on the mouse abdomen was removed with hair trimmers. After euthanasia a 1 × 1 cm^2^ patch of skin was resected. Excess fat and connective tissue were scraped off the sample. Skin was incubated in a HBSS solution containing 5 mM EDTA, 10 mM HEPES, and 10% FBS for 30 min at 37°C. Skin was then placed in HBSS media supplemented with 0.7 mg/mL collagenase (Sigma Aldrich) and cut into small pieces with surgical scissors. The tissue was incubated for 30 min at 37°C, vigorously vortexed, and single cells were isolated through a 70 μm filter (Thermo Fisher Scientific). Isolated cells were further purified by density gradient centrifugation on Ficoll (GE Healthcare) prior to further processing.

### Flow Cytometry

Single cells from lymph nodes, spleen and skin from mice randomly-selected for euthanasia in all treatment groups were treated with Mouse BD Fc block (BD Pharmigen) for 5 min to reduce nonspecific antibody binding. Cells were incubated with fluorochrome-labeled antibodies at the manufacturer's recommended dilutions for 30 min. Cells were then permeabilized for internal staining with FoxP3/Transcription Factor Staining Buffer Set (eBioscience) for nuclear proteins according to manufacturer's protocols. All antibodies were obtained from BD Pharmigen unless specified otherwise. DC were characterized as CD11c+ (clone REA754; Miltenyi Biotech) and CD45+ (clone 30-F11). Tregs were characterized as CD4+ (clone RM4-5), CD25+ (clone PC61), and FoxP3+ (Internal nuclear, clone FJK-16s) (eBioscience). Bregs were characterized as B220+ (clone RA3-6B2), CD19+ (clone 1D3), CD1d+ (clone 1B1), and CD5+ (clone 53-7.3). Elsewhere, we (20) and others (51–56) have shown that the precursor B-cells to IL-10-producing Bregs as well as >50% of IL-10 actively-producing Bregs lie inside this CD1d+ CD5+ B-cell population. Appropriate fluorochrome and antibody type matched isotypes were used, as well as single stain controls for cytometer compensation. Cytometry was conducted in the BD Influx cell sorter with 50,000 events recorded per sample. The data were analyzed with FlowJo software version 10.7.1. Data presented exclude cellular debris and are displayed as a percentage of target cells within the total intact cell population.

### Incidence of Hyperglycemia in Mice *in vivo* and Insulitis Scoring

Female NOD mice were randomly distributed into saline, PLGA-Ni, or Ins-RT-NP treatment groups with an *n* = 12 animals per group. Microparticles or vehicle were administered on Day 0, 3, 7, 14, and 21. Blood glucose was monitored twice a week. An animal was considered diabetic if two consecutive readings, spaced 2 days apart were ≥300 mg/dL. Animals were euthanized within 4 days of diabetes confirmation. Tissues collected from euthanized mice included PLN, MLN, pancreas, and spleen. To assess the grade of insulitis in randomly-selected sections of pancreata from diabetic mice as well as RT-NP recipients who were diabetes-free at 33 weeks of age, we visualized hematoxylin/eosin-stained sections by light microscopy at 10X magnification and assigned scores as follows: 1 = up to 25% of the islet mass infiltrated; 2 = between 25 and 50% of the islet infiltrated; 3 = between 50 and 75% of the islet infiltrated; and 4 = Between 75 and 100% of the islet infiltrated. Scoring was conducted in a blinded manner (i.e., pathologist was not aware of the treatment assigned to the mouse from which the sections of the pancreas were derived).

### Statistical Analyses of the Data

Statistical analysis was performed with GraphPad Prism software version 7.0c. Student's *t*-tests, ANOVA, and Kruskal-Wallis tests were performed as appropriate to compare the statistical relevance of differences in outcomes between and among treatment groups. A *p*-value of ≤ 0.05 was considered significant. Survival curves (Log-Rank test, Mantel-Cox) were analyzed for significance between two treatments at a time, where a *p* < 0.0167 was deemed significant (Bonferroni correction for three groups). Graphs are displayed as mean and standard deviation. A Dixon q test was performed to remove any data point outliers.

## Results

### TGFβ1 and Human Insulin B9-23 Peptide Bind Efficiently to PLGA-Ni Microparticles

**Figure 1:**
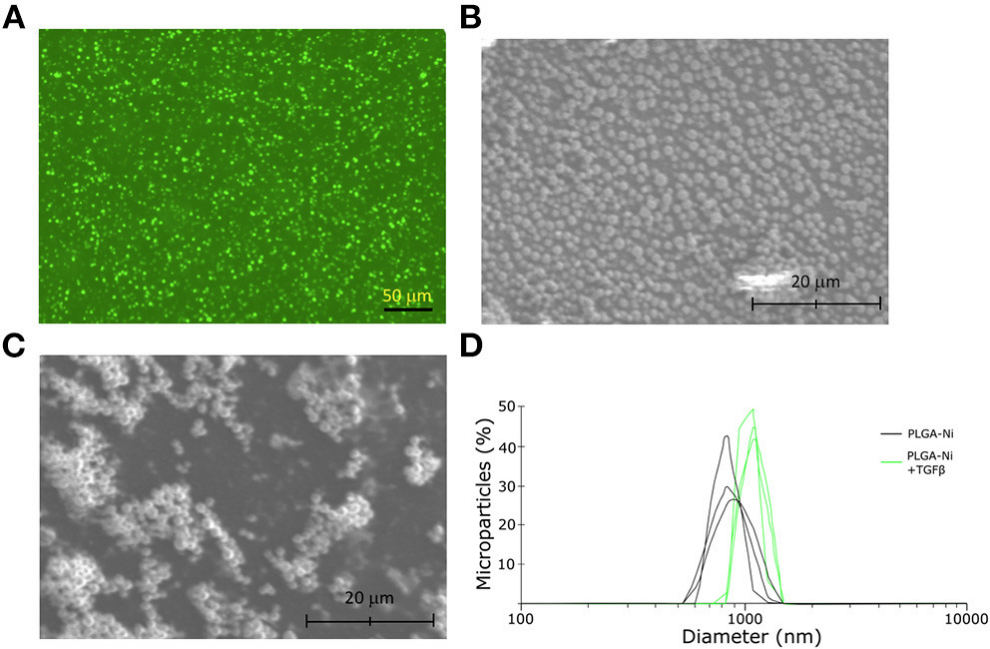
Physical characteristics of the PLGA-Ni microparticles. **(A)** Fluorescence microscopy image of His-tagged GFP admixed with PLGA-Ni microparticle suspensions. Autofluorescence has been subtracted from the image. Scanning electron microscopy images of PLGA-Ni without **(B)** and with **(C)** adsorbed TGFβ1. **(D)** A histogram of hydrodynamic microparticle size of PLGA-Ni alone or with His-tagged TGFβ1, reveals hydrodynamic diameters (D_h_) of 867.5 ± 41.6 nm (PDI = 0.18) and 1,088.6 ± 15.6 nm (PDI = 0.15), respectively.

**Figure 2:**
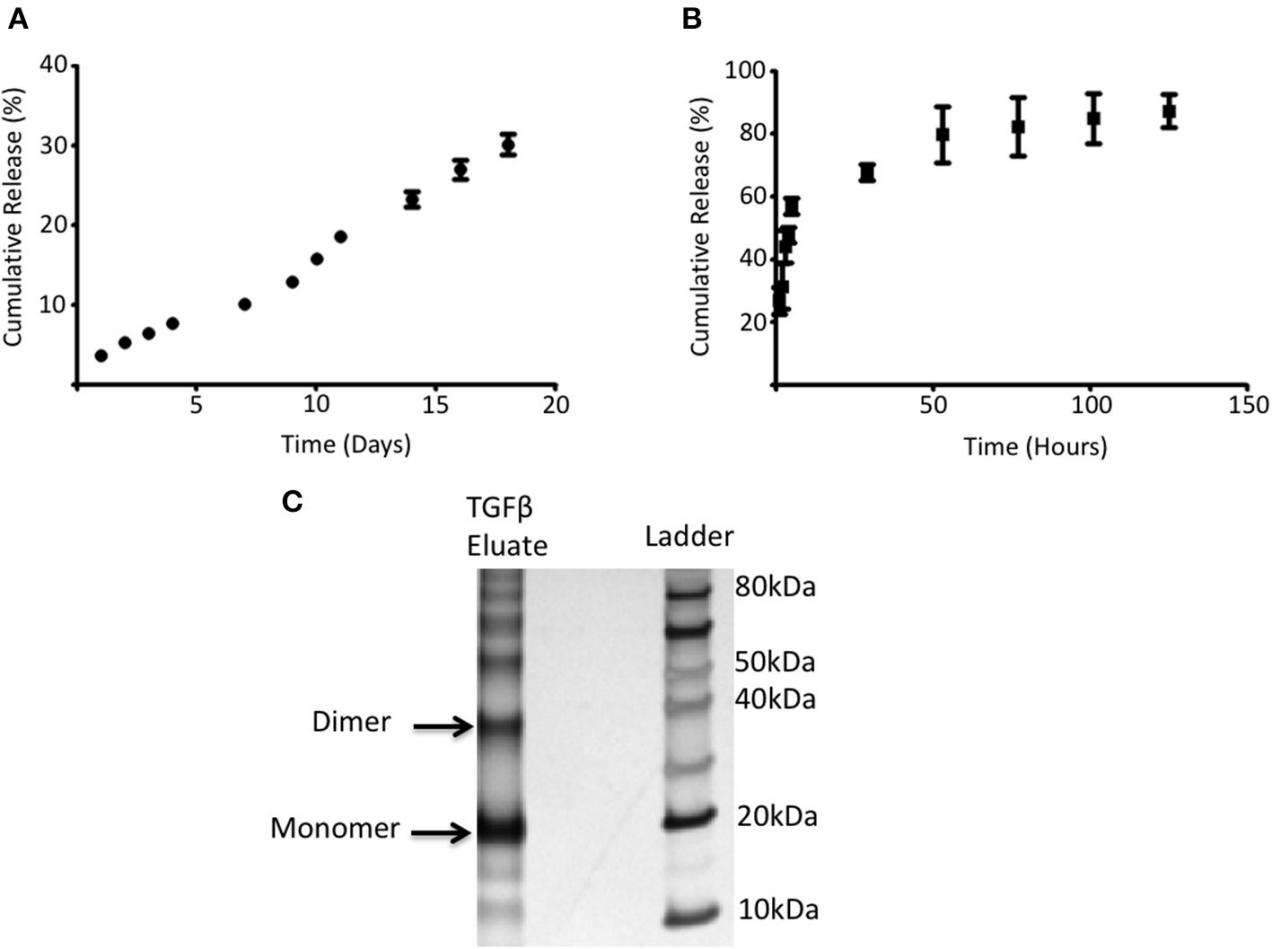
RA and adsorbed protein release from PLGA-Ni microparticles. **(A)** The rate of RA release was determined by UV absorption and shown as cumulative release (% of starting amount) over time. **(B)** 5 mg of PLGA-Ni particles with adsorbed His-tagged GFP was incubated in pH 8 Tris buffer at 37°C for 5 days. Free His-tagged GFP was measured with a fluorescent spectrometer (λ = 508 nm) at the indicated time points and the amount of free protein is shown as cumulative release (% of starting amount) over time. **(C)** Eluate from TGFβ1-adsorbed PLGA-Ni microparticles contains monomers, dimers, and higher order multimers of TGFβ1.

The ability of PGLA-Ni microparticles to adsorb His-tagged proteins was confirmed by fluorescent microscopy. Figure 1A shows His-tagged GFP conjugated PLGA-Ni microparticles. PLGA-Ni microparticles were also imaged in scanning electron microscopy where they exhibited spherical morphology (Figure 1B), and self-association when formulated as His-tagged TGFβ1 microparticles (Figure 1C). In the absence of TGFβ1, the particles are found in the micron size range with a hydrodynamic diameter (D_h_) of 867.5 ± 41.6 nm (PDI = 0.18; Figure 1D). Adding TGFβ1 to the particles increased the D_h_ to 1,088.6 ± 15.6 nm (PDI = 0.15). The kinetics of RA release was subsequently characterized *in vitro*. RA was found to release gradually from PLGA-Ni microparticles at 37°C in PBS with an average release rate of 1 μg/day from 10 mg of microparticles over 10 days *in vitro*, resulting in a total release of approximately 15 μg by day 18 at 37°C. This represents 31% of the total RA contained in 10 mg of microparticles (Figure 2A). PLGA-Ni microparticles incubated with His-tagged GFP (as a protein surrogate for TGFβ1) showed a more rapid and complete release of GFP (Figure 2B). This is indicative of the His-tag more readily detaching from the Ni ion compared to RA released from the intact or deformulating particle. GFP concentration was maintained at 150 ng/mL, yielding a concentration above the anticipated physiologically-active concentration of TGFβ1 (57). Additional preparations of PLGA-Ni microparticles were incubated with His-tagged TGFβ1 (a 17.3 kDa non-glycosylated fragment fused to a 4.5 kDa amino terminal hexa-histidine tag) and subsequently eluted. Figure 2C shows a representative result following SDS-PAGE of eluates *in vitro* from the TGFβ1-formulated microparticles indicating that upon elution, the TGFβ1 protein exists predominately as dimers and monomers, with some higher order oligomers.

### DC Take up PLGA-Ni Particles *in vitro* as Well as *in vivo*, Following Subcutaneous Administration

**Figure 3:**
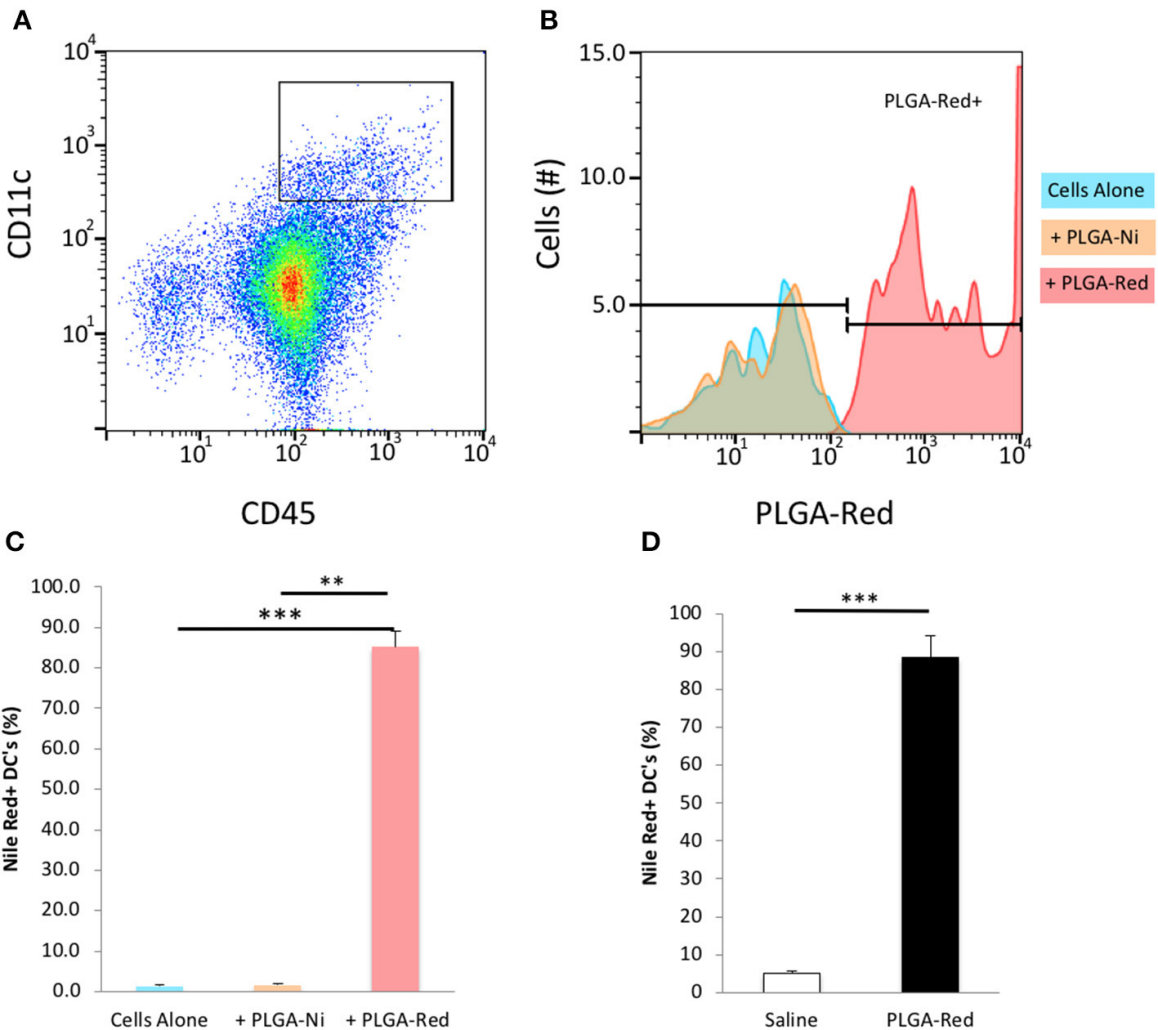
Microparticle uptake by DC *in vitro* and *in vivo*. Single cell suspensions of freshly isolated splenocytes were incubated with PLGA-Ni, PLGA-Red, or saline vehicle *in vitro* for 18 h. **(A)** Flow cytometry-identified CD45+ CD11c+ splenic DC were evaluated **(B)** for PLGA-Red microparticle uptake with PE fluorochrome settings. Saline (blue) and PLGA-Ni (orange) treatment populations showed similar PE signal indicating PLGA-Ni alone had low-to-no autofluorescence. PLGA-Red (red) treated DC showed PE signal which when quantified **(C)** displayed a 85% uptake of microparticles (*n* = 10 wells). PLGA-Red uptake was significant compared to the other treatment groups (Kruskal-Wallis test) with *p*-values of 0.0001 (saline vs. PLGA-Red) and 0.0026 (PLGA-Ni vs. PLGA-Red). PLGA-Red particles alone did not exhibit signal in DC marker fluorochrome channels (data not shown). **(D)** PLGA-Red microparticles were administered subcutaneously in mice and DC were isolated from a 1 cm^2^ patch of skin 18 h later. DC from the skin patch of PLGA-Red treated animals exhibited a 88.5% uptake of microparticles (*n* = 5 mice, Student *t*-test *p* = 0.0001). Experiments were performed twice in mice from separate NOD cohorts. Statistical significance is designated with ** if *p* < 0.01 and *** if *p* = 0.0001.

We first sought to confirm that the PLGA-Ni backbone would be taken up by DC *in vitro*. CD45+ CD11c+ DC were identified by flow cytometry (Supplementary Figure 1) in freshly-obtained splenocytes after a 18 h exposure to PLGA-Ni or PLGA-Red microparticles. In Figures 3A–C we show that Nile Red dye was detected in up to 85% of CD45+ CD11c+ DC incubated with PLGA-Red microparticles, with little detection in cell unexposed to the PLGA-Red microparticles or cells incubated with PLGA-Ni microparticles that were not formulated with Nile Red. We then sought to determine the uptake of PLGA-Red by DC in the skin following subcutaneous (s.c.) injection into 8–10 week-old female NOD mice. In Figure 3D, we show substantial accumulation (>88%) of PLGA-Red microparticles in CD45+ CD11c+ DC recovered from a 1 × 1 cm^2^ of abdominal skin (that contains the dermal and subdermal tissue; anatomic site known to facilitate the accumulation of exogenously-administered DC and microparticles inside the PLN (20, 21, 58–61) 18 h following the injection of PLGA-Red. Any fluorescence detected in CD45+ CD11c+ cells from vehicle-treated animals represents the expected autofluorescence of DC.

### Insulin B9-23 Peptide-Decorated RT-NP Treatment Prevents the Onset of Diabetes When Administered Into Early Mid-stage Insulitic NOD Mice

**Figure 4:**
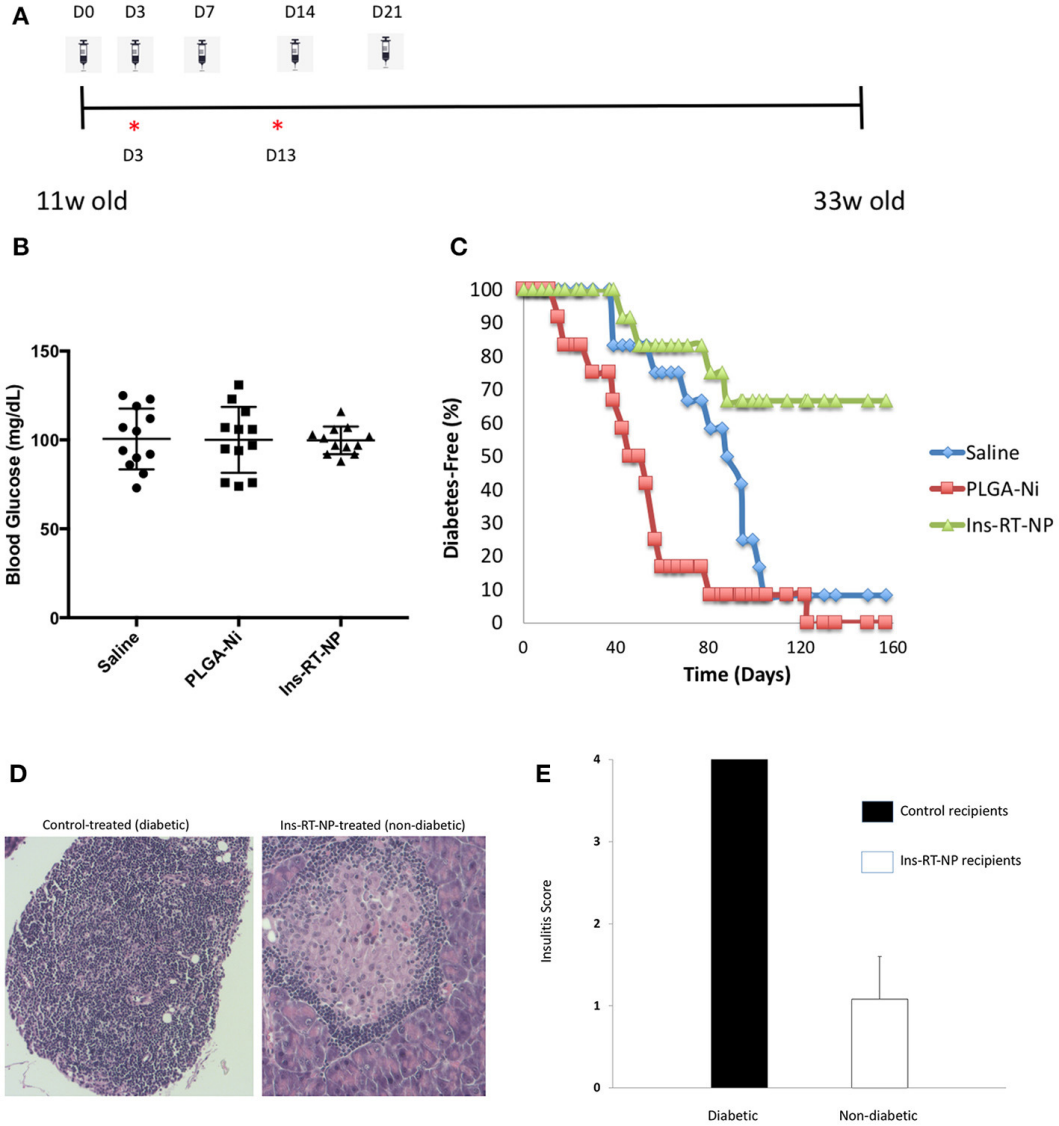
Prevention of diabetes onset following Ins-RT-NP administration. Non-diabetic female NOD mice at 11 weeks of age were randomly assigned to three treatment groups (*n* = 12 per group). Each mouse received five subcutaneous abdominal injections of saline vehicle, 10 mg of PLGA-Ni in solution, or 10 mg of Ins-RT-NP in solution as shown in the dosing schedule timeline **(A)**. The timeline also shows the tissue harvest time points (“*”) from an additional cohort of animals (“*acute study cohort*”; Figure 5). **(B)** No significant difference was observed in the starting blood glucose values (prior to microparticle injection; *p* = 0.981, Kruskal-Wallis test). **(C)** Mice were assessed for diabetes status by measuring tail vein blood glucose, where two consecutive readings over 300 mg/dL indicated diabetes onset. Shown in the graph are the survival curves representing diabetes-free mice. Diabetes incidence in Ins-RT-NP-treated mice was significantly lower when compared to saline (*p* = 0.0119) and PLGA-Ni (*p* = 0.0001) treatment. A log-rank test (Mantel-Cox) confirms significance in the differences among the treatment groups (*p* < 0.0167; *p* < 0.05 Bonferonni-corrected for three groups). **(D)** Insulitis grade was significantly-lower in pancreas sections of 33 week-old diabetes-free RT-NP recipients compared to all the diabetic mice in the control arms. Shown are images at 10X magnification representative of >5 islets/field visualized per treatment arm. **(E)** Summary of insulitis scores between all diabetic mice and the Ins-RT-NP recipients who were diabetes-free at 33 weeks of age.

Having confirmed that PLGA-Ni delivery resulted in substantial accumulation inside DC, with the expectation that these DC would be converted into tolerogenic cells using RA and TGFβ1-formulated microparticles, we then sought to determine if a PLGA-Ni formulation of RA and TGFβ1 additionally-engineered with adsorbed insulin peptide B9-23 (Ins-RT-NP) could alter the progression of the underlying autoimmunity in NOD female mice toward overt diabetic hyperglycemia. In Figure 4A, we show the timeline of microparticle injections, where each of the mice were administered five s.c. injections of either vehicle saline, PLGA-Ni, or Ins-RT-NP starting at 11 weeks of age. This is an age where insulitis is evident inside the pancreas of NOD mice. Prior to randomization into the treatment arms, NOD female mice between 9 and 11 weeks of age were pre-screened to insure absence of hyperglycemia. Fasting blood glucose, immediately prior to treatment, did not exceed 131 mg/dL (Figure 4B). In Figure 4C, we show that Ins-RT-NP administration beginning at 11 weeks of age when substantial insulitis is known to exist, prevented the onset of hyperglycemia in a significant proportion of recipients compared to mice treated with microparticles that were devoid of RA, TGFβ1 and insulin B9-23 peptide, or mice treated with vehicle alone. It is noteworthy that the diabetes-free state was maintained for a substantial amount of time (33 week-old mice; 22 weeks diabetes-free following treatment) without additional injections of the microparticles. Insulitis grade was significantly-lower in pancreas sections of 33 week-old diabetes-free RT-NP recipients compared to all the diabetic mice in the control arms (Figures 4D,E).

### Increased Frequency of Bregs but Not Foxp3+ Tregs in Ins-RT-NP-Treated NOD Mice

**Figure 5:**
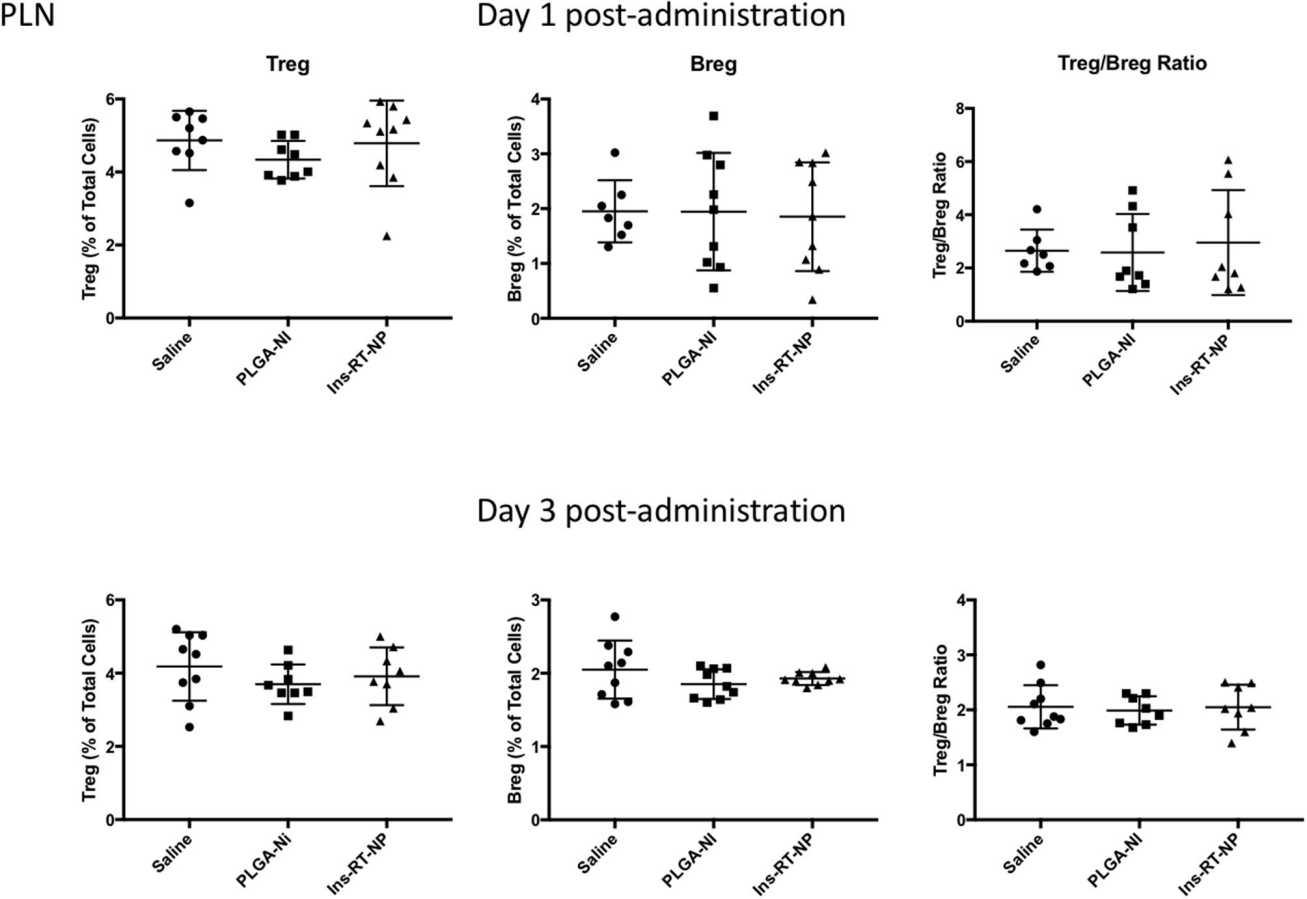
Changes in the frequency of regulatory lymphocyte cell populations inside the PLN of NOD mice following acute microparticle administration. Animals from the acute study cohort (See timeline in Figure 4A) were euthanized at 1 or 3 days following test article administration. Single cell suspensions from resected PLN were then assessed by flow cytometry for Treg and Breg frequency. There were no discernible differences in the Treg or Breg frequency in the PLN among the mice of the three treatment arms at 1 or 3 days following microparticle administration. Similarly the ratios between Treg and Breg cells were unaltered. Data are combined from the outcomes in two independent animal cohorts examined at different times in the study (*n* = 7–9 per arm).

**Figure 6:**
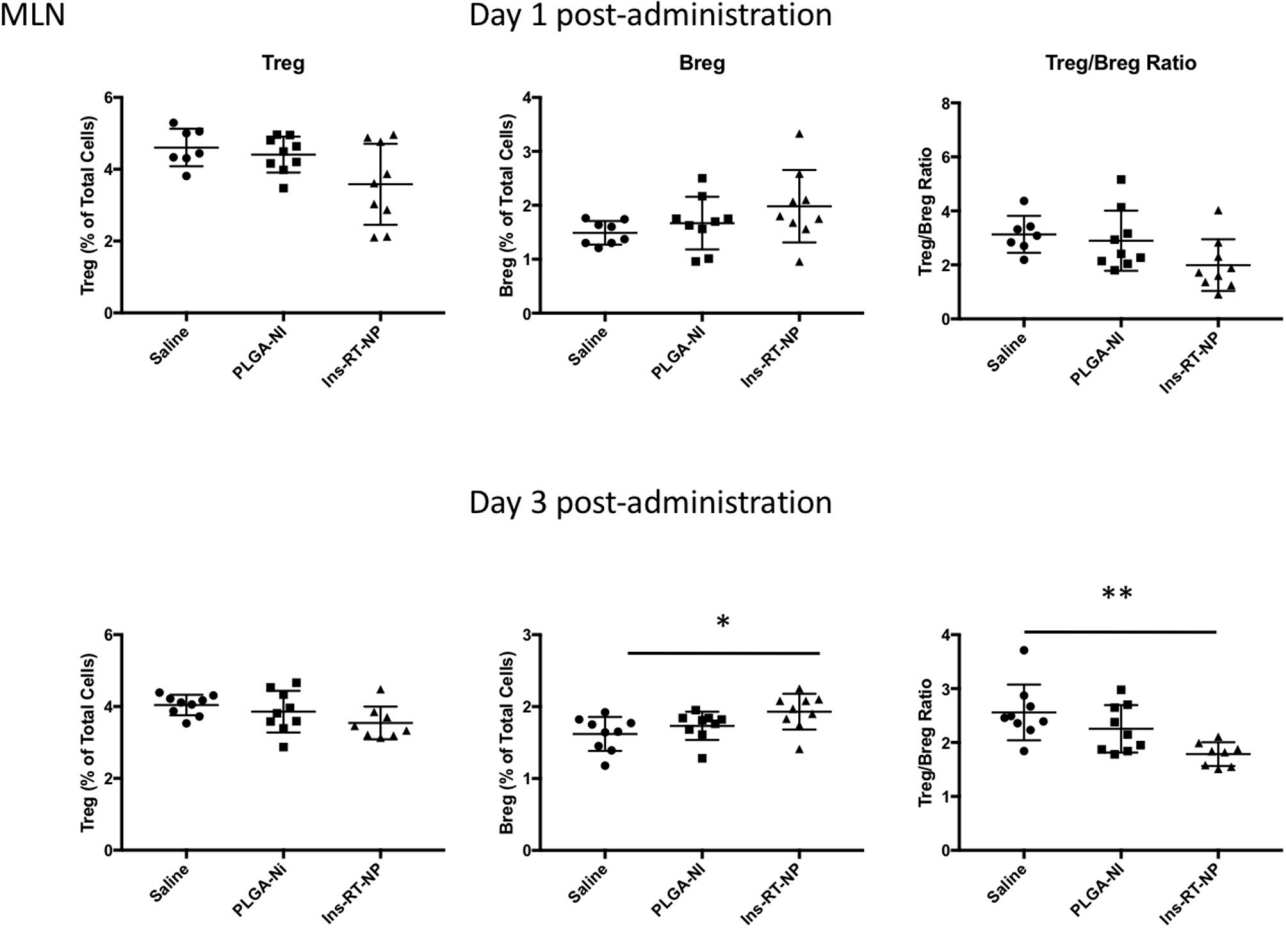
Changes in the frequency of regulatory lymphocyte cell populations inside the MLN of NOD mice following acute microparticle administration. Animals from the acute study cohort (See timeline in Figure 4A) were euthanized at 1 or 3 days following test article administration. Single cell suspensions from resected MLN were then assessed by flow cytometry for Treg and Breg frequency. A non-significant downward trend is seen in Treg frequency in MLN of mice treated with Ins-RT-NP vs. saline. Breg frequency exhibited an upward trend in mice treated with Ins-RT-NP, becoming statistically-significant significant compared to control test articles by the time of the third injection (*n* = 9, ANOVA *p* = 0.0262). Tukey's multiple comparisons determined *p* = 0.0215 between the saline and Ins-RT-NP treatment group. The Treg/Breg ratio was similarly significantly-different with the three-injection treatment (ANOVA *p* = 0.0035, post test *p* = 0.0025 for saline vs. Ins-RT-NP). Statistical significance is displayed as * for *p* < 0.05 and ** for *p* < 0.01 as assessed by ANOVA. Data are combined from the outcomes in two independent animal cohorts examined at different times in the study.

Data by others indicate that nanoparticle formulations of RA and TGFβ can convert T-cells into Tregs (37), and when administered (nanoparticles or hydrogels) into NOD female mice, they foster an increase in the number of Foxp3+ Tregs inside the PLN and/or the pancreata of diabetes-free mice (58, 59, 62, 63). Since there were no surviving mice in the control treatment arms at 22 weeks following test article administration, it proved challenging for us to interpret the differences in Tregs in PLN and MLN obtained from vehicle or PLGA-Ni-treated mice to those in the diabetes-free Ins-RT-NP recipients. Therefore, to ensure matched populations and matched times at which PLN and MLN were collected in order to obtain interpretable data, we used an acute model of test article administration. For this, 11 week-old NOD mice in all treatment arms (*n* = 7–9) were euthanized 1 or 3 days following microparticle or vehicle administration. We then measured regulatory immune cell population numbers by flow cytometry (Supplementary Figures 2, 3) in PLN and MLN from these mice. Much to our surprise, and in contrast to our expectations, the frequency of Foxp3+ Tregs inside the PLN among all treatment groups was not different (Figure 5). There was a surprising lower, albeit statistically-not significant, Treg content in the MLN of mice treated with Ins-RT-NP compared to the controls (Figure 6). Considering the possibility that the viability of the cells inside the pancreatic and/or mesenteric lymph nodes could be affected by microparticles drained into the tissue (PLN and/or MLN), we measured the viability of single cells from dispersed lymph node tissue. We did not see any significant differences in viability of single cells from PLN and MLN of diabetes-free mice (Supplementary Figure 4). We then looked for possible differences in Breg frequency. Although we could not discern any differences inside the PLN of mice among the three treatment arms (Figure 5), we confirmed a small but statistically-relevant difference inside the MLN of mice treated with the Ins-RT-NP (Figure 6) at 3 days following administration. The ratio of Tregs to Bregs in PLN at 1 or 3 days following test article administration was statistically-indistinguishable among the treatment arms (Figure 5). In the MLN, however, the ratio was statistically-different in the Ins-RT-NP-treatment arm at 3 days post-administration compared to the other treatments (Figure 6).

## Discussion

Although insulin therapy can adequately manage the day-to-day glucose control in T1D, the lifestyle changes, the considerable risk of insulin-induced hypoglycemia, and the progression of many T1D patients to cardiovascular, renal, neural, and ophthalmic complications, indicate that pharamcologic insulin is not a cure. The once widely-held dogma that insulin-requiring patients were devoid of a substantial mass of beta cells inside the pancreas due to autoimmune destruction, has now yielded to a more balanced view. Based on accumulating evidence, a respectable mass of beta cells remains in T1D patients whose disease duration can be long-standing (64–69) that is functionally-impaired, although potentially “re-activatable” if the underlying autoimmunity as well as non-autoimmune pancreas-selective innate inflammation can be attenuated. A number of approaches have been implemented over the past decade to depress the underlying autoimmunity including single or combination agents from the categories of pharmacologic immunosuppressives, cytokine neutralizing antibodies, lymphocyte depletion, and T1D autoantigens (70–80). Where animal studies demonstrated sufficient efficacy, when implemented as clinical trials, each of the referenced approaches could not achieve long-term insulin independence (70–72). In fact, systemic as well as parenteral administration of most of these agents resulted in significant toxicity and adverse effects.

Micro/nanoparticle-based delivery of immunomodulatory agents, with or without autoantigen co-delivery to treat autoimmune disease, offers many attractive advantages over systemic immunomodulators. Site-specific delivery minimizes off-target effects, licenses leukocytes that can be “reprogrammed” to build regulatory leukocyte networks that can become antigen-specific, even in the absence of exogenous supply of autoantigen(s). We have demonstrated the latter using a specific abdominal site in mice and non-human primates, whose epidermal space is served by lymphatic drainage that accumulates or transits, in part, inside the PLN (18, 21). Taking advantage of this intriguing anatomical feature, we demonstrated that autoantigen-free microsphere formulations of antisense DNA targeting the primary transcripts of CD40, CD80, and CD86, preferentially accumulated inside PLN, generated Foxp3+ Tregs therein, and in NOD mice, prevented the progression of late-stage dysglycemia to overt clinical diabetes and also achieved reversal of new-onset hyperglycemia and long-term stability of a normoglycemic state (18, 21).

Our technology (18, 21) was developed to exploit the phagocytic nature of epidermis-residing as well as migratory DC to take up exogenously-administered particles loaded with molecules that are able to direct them toward a functionally-tolerogenic state *in vivo*. Since then, other groups have developed conceptually similar architecture with different approaches to stabilize functional tolerance in DC (40, 58, 59, 81–92). These approaches have resulted in successful treatment of autoimmune disease in animal models of T1D (58, 59) and multiple sclerosis (81–83, 85). As we continue to decipher the mechanisms that our earlier-reported microspheres use to confer tolerogenic functional properties to DC beyond the knockdown of the primary transcripts of CD40, CD80, and CD86, we have uncovered the involvement of RA (19). The combination of the DNA antisense oligonucleotides stimulates the production of RA by DC in a manner that involves cell-intrinsic retinaldehyde dehydrogenase 1 (RALDH1) (19).

The combination of RA and TGFβ1 is critical in peripheral generation of immunosuppressive T-cells characterized by the expression of the Foxp3 transcription factor (Foxp3+ Tregs) (93–98). Tregs prevent emergence of autoimmune disease (99, 100). Subsets of Foxp3+ Tregs are found in mice and humans: natural Treg and induced Treg (iTreg) (101, 102). In the periphery, iTreg differentiate from naive T-cells under sub-immunogenic conditions of antigen presentation and in the presence of RA and TGFβ (103). That RA is a cofactor in the generation of iTregs stems from *in vitro* findings that mesenteric lymph node and lamina propria DC potently induced iTreg differentiation in the presence of TGFβ (98, 104, 105). Addition of RA to co-cultures with splenic DC and TGFβ enhances iTreg induction (94, 98, 105). Exogenous RA sustains iTreg generation in conditions that typically oppose it, such as presence of inflammatory cytokines (IL-6, IL-21) and high co-stimulatory environments (34, 94, 98). These observations made for a very compelling case to combine RA with TGFβ1 with a T1D-relevant autoantigen in a microparticle formulation. One could argue that non-formulated RA and TGFβ1 co-administered could achieve similar if not identical outcomes thereby questioning the need to engineer micro-/nanoparticle formulations of the agents. To our knowledge, and also in our experience, administration of RA or TGFβ1 alone or together as an injectable suspension cannot prevent T1D (unpublished observations) even though iTreg frequency is increased inside the site of the injection in the skin but not the draining lymph nodes. Also, the iTregs are not functionally-stable in suppression assays (unpublished observations). As the studies reported herein were underway, a number of supportive findings were published using particle designs different from ours. Keselowsky et al., using a mixture of two separate microparticle populations (population 1 consisting of TGFβ or IL-10 and population 2 consisting of either rapamaycin or RA), conferred a tolerogenic-reminiscent phenotype in DC *in vitro* (40). They also developed a more complex variation where one of the microparticle populations contained either GM-CSF or TGFβ and the other contained vitamin D3 and denatured insulin, to prevent progression of early-stage autoimmunity to diabetic hyperglycemia and to reverse, to some degree, new-onset hyperglycemia (59). This approach resulted in protective outcomes similar to those achieved by Miller et al. in mouse models of multiple sclerosis (81, 83, 85). Mechanistically, the involvement of DC as mediators of a tolerogenic state, mainly via the increase in Treg numbers, was a shared feature of these approaches (59, 81, 83, 85).

In addition to the increase in the frequency of Tregs, we have shown that one mechanism by which RA participates in the induction of immune hyporesponsiveness is through the differentiation of B-lymphocytes into IL-10+ Bregs as well as the proliferation of existing Bregs (19, 20). Although initial studies indicated that suppression ability was specific for IL-10+ CD1d+ CD5+ B-cells, it was later discovered that the progenitors of as well as the majority of IL-10-producing Bregs resided inside CD1d+ CD5+ population and that additionally, IL-10 expression was not a condition sine qua non for CD1d+ CD5+ B-cell suppressive ability (51–56). To date, none of the previous studies in the delivery of potentially-tolerogenic micro/nanoparticles into animal models of autoimmunity have studied their effects on Bregs as possible unique or Treg-overlapping cellular mediators of the action of the micro/nanoparticles. Our report here is the first to demonstrate the possible involvement of Bregs in the absence of increases in Treg frequency in NOD mice acutely-treated with single microparticle-formulated RA, TGFβ1 and insulin autoantigen peptide. What is, at the moment, unclear, is why Bregs are elevated inside the MLN when compared to the PLN (Figures 5, 6). Although NOD mice treated with Ins-RT-NP exhibit Foxp3 positivity at 22 weeks from the time of the last microparticle injection (data not shown), in the absence of age-matched diabetes-free and age-matched new-onset diabetic mice treated with the control test articles, it is currently not known if the frequency of Tregs or Bregs changes longitudinally resulting in a greater population of these cells inside the PLN and/or the MLN of Ins-RT-NP-treated mice. If so, the observations we present following acute administration will not reflect a mechanistically-interpretable outcome, when measured longitudinally. These studies are currently underway with large animal cohorts to capture age-matched diabetic and non-diabetic control microparticle recipients. Nevertheless, the microparticle formulation, when delivered into NOD mice is protective against the progression of mid-stage autoimmunity to overt clinical hyperglycemia.

Our Ins-RT-NP delivery system has several advantages over the other reported approaches, referenced above. First is in the design complexity. Our system consists of a *single* formulation that brings together only two bioactive agents (RA and TGFβ1) with the addition of a unique T1D-relevant autoantigen. Second, from a biologic aspect, DC need acquire only one population of particles without the potential of competition for uptake and/or selectivity in action imposed by a second population. Third, from a potential future regulatory aspect, a single population of easily-characterizable particles that are manufactured to predictable physicochemical properties facilitates a target product profile that would be human-specific compared to more complex microparticle chemistry and the requirement for more than one particle population. Finally, only a short course of microsphere treatment (five injections) was required to achieve a long-term diabetes-free state (Figure 4). It is possible that even fewer injections could achieve comparable outcomes, something that is under investigation currently. This suggests that, even if the induced Bregs are the only layer of autoimmunity regulation, their effect is potentially long lasting (e.g., due to a longitudinal expansion and/or a long half-life of these Bregs *in vivo*). Although this does not rule out the possibility that other regulatory cell changes occur in different organs or time points of the treatment progression.

Recently, an orally-delivered formulation of TGFβ and RA was reported to result in decreased potentially-autoreactive effector T-cells inside the pancreas of mice rendered diabetic by multiple low-dose streptozotocin (MLDS) (106). The reported data suggest suppressed insulitis and a statistically-greater proportion of diabetes-free mice in the RA/TGFβ microparticle treatment arm compared to controls. The conceptual novelty and achievement of an orally-available microparticle formulation is very noteworthy and certainly commendable, however, the findings raise some important questions that need to be addressed. First is the model. All studies, including ours, using micro/nanoparticle approaches to treat T1D, use the NOD/LtJ strain mouse strain, widely regarded as the best *in vivo* test system for T1D immunotherapeutics, so long as one is aware of the model's limitations (72, 107–110). The authors, instead used a model (MLDS in C57BL/6 mice) that does not reflect the genetic component as well as the chronic development of an underlying autoimmune state. Second, diabetes incidence was only monitored up to 28 days following the MLDS treatment. In our view, this time frame is insufficient to draw any conclusions about the stability of any efficacy. Third, most of the measured differences in the frequency of leukocyte populations recovered from mice in the different treatment arms in that report are not readily discernible. The differences appear to be quite small. Nevertheless, that report can be considered a first in terms of demonstrating proof-in-concept, at least in terms of oral delivery of a potentially-tolerogenic microparticle system that has a broad impact on treating autoimmunity.

Our findings support the ongoing trajectory to develop TGFβ/RA-based tolerogenic micro/nanoparticle “vaccines” for the treatment of T1D (38–40, 58, 81). The strengths of our system and approach include a simple one-population microparticle with the efficacy to prevent the progression of mid-stage autoimmunity characterized by fulminant insulitis to overt diabetic hyperglycemia achieved by only five s.c. injections of an amount of particles that would translate to a well-tolerated human-relevant target product profile. Furthermore, we have achieved long term stability of the normoglycemic state (33 weeks) that may in part be due to the regulation of the progressive autoimmunty via Bregs, at least in the MLN, even though the increase in Bregs we present herein (Figure 6) was an acute response specifically to the Ins-RT-NP. It remains to be determined if Breg frequency in diabetes-free mice remains increased in Ins-RT-NP compared to age-matched diabetic control mice (untreated and control test article-treated recipients). The differences in the Treg:Breg ratio we observe in the MLN among the treatment arms at 3 days post-administration of the Ins-RT-NP are intriguing and remain to be mechanistically-understood. It is possible that our particles act by modifying the biology of other T-cells by a number of overlapping and complementary mechanisms of action. For example, DC that take up these particles could modify the numbers and/or action of Tr1 regulatory T-cells and not Foxp3+ Tregs. Or, the surface-bound TGFβ1 on our particles could bind to TGF receptors on DC and /or other regulatory leukocytes to license their suppressive abilities that are expressed only inside secondary lymphoid organs. These possibilities, among a number of others, are currently under investigation so that we can better understand the mechanism of action of our particle system.

Whether provision of other, or additional T1D autoantigens to the TGFβ/RA-based formulation can improve the prophylactic outcomes or could reverse new-onset hyperglycemia is currently unknown, although many other particle formulations tested appear to require at least one autoantigen peptide or intact protein to induce some form of disease-specific immune hyporesponsiveness (40, 58, 59, 81–92).

## Data Availability Statement

The original contributions presented in the study are included in the article/Supplementary Material, further inquiries can be directed to the corresponding author/s.

## Ethics Statement

The animal study was reviewed and approved by AHN IACUC; Allegheny Health Network IACUC.

## Author Contributions

BP, CE, and YG administered the microparticles into the mice, collected the tissues and organs, conducted the immunophenotyping, conducted the statistical analyses, and wrote the initial version of the manuscript. WM, YW, and WL manufactured the microparticle formulations and characterized those formulations, wrote the sections in the materials and methods part of the manuscript that describe the manufacture and characterization of the particles, and verified the statistical analyses. RL and BP conducted the flow cytometry and analysis of the outcomes and participated in the statistical analysis of those data. MT and NG edited the manuscript from the first draft onwards, discussed the findings and the significance with the co-authors, and verified the statistical outcomes. NG as principal investigator of the study was responsible for the oversight of the study at all levels and ensured that the outcomes were reproducible, that the experiments were rigorously conducted, and that the data presented in this manuscript reflect the raw data obtained faithfully.

## Conflict of Interest

The authors declare that the research was conducted in the absence of any commercial or financial relationships that could be construed as a potential conflict of interest.

## Supplementary Material

Supplementary Figure 1 Dendritic cell antibody isotypes and controls used in flow cytometry. Single cell suspensions of splenocytes were isolated and grown *in vitro*. Splenocytes were analyzed by flow cytometry to identify dendritic cells using the CD45 and CD11c cell surface markers 18 h later. Fluorescence histograms of unstained cells are shown in red and cells stained with matched fluorochrome-conjugated isotypes are shown in blue. Cells with antibody against **(A)** CD45 or **(B)** CD11c are shown in orange. A signal intensity cut off is shown which displays positively stained cells to the right. **(C)** Signal intensity is shown in both CD45 and CD11c channels, with CD45+ CD11c+ cells located inside the box.

Supplementary Figure 2 Antibody controls and gating method in Treg flow cytometry. Single cell suspensions of lymph nodes were analyzed by flow cytometry to identify Tregs by cell surface markers CD4, CD25, and nuclear marker FoxP3. Histograms of control unstained cells are shown in red and cells stained with matched fluorochrome-conjugated isotypes are shown in blue. Cells with antibody against **(A)** CD4, **(B)** CD25, **(C)** FoxP3 are shown in orange. A signal intensity cut off is shown which displays positively stained cells to the right. **(D)** The gating method for identifying Foxp3+ Tregs is shown. First, cells are selected for low granularity based on low side scatter. Within this population, CD4+ cells are selected as a general T-cell population. Finally, within the CD4+ cell population, cells that are CD25+ FoxP3+ are selected for further analysis and quantitation.

Supplementary Figure 3 Breg antibody controls and gating method in Breg flow cytometry. Single cell suspensions of lymph nodes were analyzed by flow cytometry to identify Bregs by cell surface markers CD19, B220, CD1d, and CD5. Histograms of control unstained cells are shown in red and cells stained with matched fluorochrome-conjugated isotypes are shown in blue. Cells with antibody against **(A)** CD19, **(B)** B220, **(C)** CD1d, and **(D)** CD5 are shown in orange. A signal intensity cut off is shown which displays positively stained cells to the right. **(E)** The gating method for identifying Bregs is shown. First, cells are selected for low granularity as indicated by low side scatter. Within this population, the prevalent CD19+ B220+ double positive B-cells are selected. Finally, within this B cell population, cells that are CD1d+ CD5+ are selected as shown for further analysis.

Supplementary Figure 4 Lymph node (LN) cell viability. Animals received a single injection of saline, 10 mg PLGA-Ni, or 10 mg Ins-RT-NP. Single cell suspensions were made from MLN and PLN. Cell viability was assessed after LN dissociation by Trypan Blue staining. The percentage of viable cells is displayed and no significant differences in viability were detected (individual one-way ANOVA per LN type). Experiments include two separate animal cohorts combined (*n* = 9).

## References

[1] Insel RA Dunne JL Atkinson MA Chiang JL Dabelea D Gottlieb PA. Staging presymptomatic type 1 diabetes: a scientific statement of JDRF, the Endocrine Society, and the American Diabetes Association. Diabetes Care. (2015) 38:1964–74. 10.2337/dc15-1419 26404926 PMC5321245

[2] Atkinson MA Eisenbarth GS Michels AW. Type 1 diabetes. Lancet. (2014) 383:69–82. 10.1016/S0140-6736(13)60591-7 23890997 PMC4380133

[3] Atkinson MA. The pathogenesis and natural history of type 1 diabetes. Cold Spring Harb Perspect Med. (2012) 2. 10.1101/cshperspect.a007641 23125199 PMC3543105

[4] Roep BO Atkinson M von Herrath M. Satisfaction (not) guaranteed: re-evaluating the use of animal models of type 1 diabetes. Nat Rev Immunol. (2004) 4:989–97. 10.1038/nri1502 15573133

[5] Emamaullee JA Davis J Merani S Toso C Elliott JF Thiesen A. Inhibition of Th17 cells regulates autoimmune diabetes in NOD mice. Diabetes. (2009) 58:1302–11. 10.2337/db08-1113 19289457 PMC2682686

[6] Ferraro A Socci C Stabilini A Valle A Monti P Piemonti L. Expansion of Th17 cells and functional defects in T regulatory cells are key features of the pancreatic lymph nodes in patients with type 1 diabetes. Diabetes. (2011) 60:2903–13. 10.2337/db11-0090 21896932 PMC3198077

[7] Jain R Tartar DM Gregg RK Divekar RD Bell JJ Lee HH. Innocuous IFNgamma induced by adjuvant-free antigen restores normoglycemia in NOD mice through inhibition of IL-17 production. J Exp Med. (2008) 205:207–18. 10.1084/jem.20071878 18195074 PMC2234380

[8] Wang B Andre I Gonzalez A Katz JD Aguet M Benoist C. Interferon-gamma impacts at multiple points during the progression of autoimmune diabetes. Proc Natl Acad Sci USA. (1997) 94:13844–9. 10.1073/pnas.94.25.13844 9391115 PMC28395

[9] Sakaguchi S Ono M Setoguchi R Yagi H Hori S Fehervari Z. Foxp3+ CD25+ CD4+ natural regulatory T cells in dominant self-tolerance and autoimmune disease. Immunol Rev. (2006) 212:8–27. 10.1111/j.0105-2896.2006.00427.x 16903903

[10] Miska J Abdulreda MH Devarajan P Lui JB Suzuki J Pileggi A. Real-time immune cell interactions in target tissue during autoimmune-induced damage and graft tolerance. J Exp Med. (2014) 211:441–56. 10.1084/jem.20130785 24567447 PMC3949570

[11] Salvany-Celades M van der Zwan A Benner M Setrajcic-Dragos V Bougleux Gomes HA Iyer V. Three types of functional regulatory T cells control T cell responses at the human maternal-fetal interface. Cell Rep. (2019) 27:2537–47 e5. 10.1016/j.celrep.2019.04.109 31141680

[12] Wu J Ma S Hotz-Wagenblatt A Angel P Mohr K Schlimbach T. Regulatory T cells sense effector T-cell activation through synchronized JunB expression. FEBS Lett. (2019) 593:1020–9. 10.1002/1873-3468.13393 31017652

[13] Golab K Krzystyniak A Marek-Trzonkowska N Misawa R Wang LJ Wang X. Impact of culture medium on CD4(+) CD25(high)CD127(lo/neg) Treg expansion for the purpose of clinical application. Int Immunopharmacol. (2013) 16:358–63. 10.1016/j.intimp.2013.02.016 23466550

[14] Marek-Trzonkowska N Mysliwec M Siebert J Trzonkowski P. Clinical application of regulatory T cells in type 1 diabetes. Pediatr Diabetes. (2013) 15:322–32. 10.1111/pedi.12029 23627860

[15] Thompson JA Perry D Brusko TM. Autologous regulatory T cells for the treatment of type 1 diabetes. Curr Diab Rep. (2012) 12:623–32. 10.1007/s11892-012-0304-5 22843491

[16] Bluestone JA Buckner JH Fitch M Gitelman SE Gupta S Hellerstein MK. Type 1 diabetes immunotherapy using polyclonal regulatory T cells. Sci Transl Med. (2015) 7:315ra189. 10.1126/scitranslmed.aad4134 26606968 PMC4729454

[17] Marek-Trzonkowska N Mysliwiec M Dobyszuk A Grabowska M Derkowska I Juscinska J. Therapy of type 1 diabetes with CD4(+)CD25(high)CD127-regulatory T cells prolongs survival of pancreatic islets - results of one year follow-up. Clin Immunol. (2014) 153:23–30. 10.1016/j.clim.2014.03.016 24704576

[18] Phillips B Nylander K Harnaha J Machen J Lakomy R Styche A. A microsphere-based vaccine prevents and reverses new-onset autoimmune diabetes. Diabetes. (2008) 57:1544–55. 10.2337/db07-0507 18316361 PMC2713034

[19] Di Caro V Phillips B Engman C Harnaha J Trucco M Giannoukakis N. Retinoic acid-producing, ex-vivo-generated human tolerogenic dendritic cells induce the proliferation of immunosuppressive B lymphocytes. Clin Exp Immunol. (2013) 174:302–17. 10.1111/cei.12177 23865694 PMC3828834

[20] Di Caro V Phillips B Engman C Harnaha J Trucco M Giannoukakis N. Involvement of suppressive B-lymphocytes in the mechanism of tolerogenic dendritic cell reversal of type 1 diabetes in NOD mice. PLoS ONE. (2014) 9:e83575. 10.1371/journal.pone.0083575 24465383 PMC3894962

[21] Engman C Wen Y Meng WS Bottino R Trucco M Giannoukakis N. Generation of antigen-specific Foxp3+ regulatory T-cells *in vivo* following administration of diabetes-reversing tolerogenic microspheres does not require provision of antigen in the formulation. Clin Immunol. (2015) 160:103–23. 10.1016/j.clim.2015.03.004 25773782

[22] Barbi J Pardoll D Pan F. Treg functional stability and its responsiveness to the microenvironment. Immunol Rev. (2014) 259:115–39. 10.1111/imr.12172 24712463 PMC3996455

[23] Chaudhry A Rudensky AY. Control of inflammation by integration of environmental cues by regulatory T cells. J Clin Invest. (2013) 123:939–44. 10.1172/JCI57175 23454755 PMC3582113

[24] Hoeppli RE Wu D Cook L Levings MK. The environment of regulatory T cell biology: cytokines, metabolites, the microbiome. Front Immunol. (2015) 6:61. 10.3389/fimmu.2015.00061 25741338 PMC4332351

[25] Pesenacker AM Broady R Levings MK. Control of tissue-localized immune responses by human regulatory T cells. Eur J Immunol. (2015) 45:333–43. 10.1002/eji.201344205 25378065

[26] Konkel JE Zhang D Zanvit P Chia C Zangarle-Murray T Jin W. Transforming growth factor-beta signaling in regulatory T cells controls T helper-17 cells and tissue-specific immune responses. Immunity. (2017) 46:660–74. 10.1016/j.immuni.2017.03.015 28423340 PMC12230991

[27] Oh SA Liu M Nixon BG Kang D Toure A Bivona M. Foxp3-independent mechanism by which TGF-beta controls peripheral T cell tolerance. Proc Natl Acad Sci USA. (2017) 114:E7536–44. 10.1073/pnas.1706356114 28827353 PMC5594672

[28] Tran DQ. TGF-beta: the sword, the wand, and the shield of FOXP3(+) regulatory T cells. J Mol Cell Biol. (2012) 4:29–37. 10.1093/jmcb/mjr033 22158907

[29] Wan YY Flavell RA. 'Yin-Yang' functions of transforming growth factor-beta and T regulatory cells in immune regulation. Immunol Rev. (2007) 220:199–213. 10.1111/j.1600-065X.2007.00565.x 17979848 PMC2614905

[30] Wan YY Flavell RA. Regulatory T cells, transforming growth factor-beta, immune suppression. Proc Am Thorac Soc. (2007) 4:271–6. 10.1513/pats.200701-020AW 17607012 PMC2647629

[31] Wrzesinski SH Wan YY Flavell RA. Transforming growth factor-beta and the immune response: implications for anticancer therapy. Clin Cancer Res. (2007) 13(18 Pt 1):5262–70. 10.1158/1078-0432.CCR-07-1157 17875754

[32] Xu A Liu Y Chen W Wang J Xue Y Huang F. TGF-beta-induced regulatory T cells directly suppress B cell responses through a noncytotoxic mechanism. J Immunol. (2016) 196:3631–41. 10.4049/jimmunol.1501740 27001954 PMC4868785

[33] Zheng SG Gray JD Ohtsuka K Yamagiwa S Horwitz DA. Generation ex vivo of TGF-beta-producing regulatory T cells from CD4+CD25- precursors. J Immunol. (2002) 169:4183–9. 10.4049/jimmunol.169.8.4183 12370347

[34] Xiao S Jin H Korn T Liu SM Oukka M Lim B. Retinoic acid increases Foxp3+ regulatory T cells and inhibits development of Th17 cells by enhancing TGF-beta-driven Smad3 signaling and inhibiting IL-6 and IL-23 receptor expression. J Immunol. (2008) 181:2277–84. 10.4049/jimmunol.181.4.2277 18684916 PMC2722959

[35] Zunino SJ Storms DH Stephensen CB. Diets rich in polyphenols and vitamin A inhibit the development of type I autoimmune diabetes in nonobese diabetic mice. J Nutr. (2007) 137:1216–21. 10.1093/jn/137.5.1216 17449584

[36] Stosic-Grujicic S Cvjeticanin T Stojanovic I. Retinoids differentially regulate the progression of autoimmune diabetes in three preclinical models in mice. Mol Immunol. (2009) 47:79–86. 10.1016/j.molimm.2008.12.028 19211146

[37] Capurso NA Look M Jeanbart L Nowyhed H Abraham C Craft J. Development of a nanoparticulate formulation of retinoic acid that suppresses Th17 cells and upregulates regulatory T cells. Self Nonself. (2010) 1:335–40. 10.4161/self.1.4.13946 21487509 PMC3062389

[38] Keijzer C Spiering R Silva AL van Eden W Jiskoot W Vervelde L. PLGA nanoparticles enhance the expression of retinaldehyde dehydrogenase enzymes in dendritic cells and induce FoxP3(+) T-cells *in vitro*. J Control Release. (2013) 168:35–40. 10.1016/j.jconrel.2013.02.027 23500056

[39] McHugh MD Park J Uhrich R Gao W Horwitz DA Fahmy TM. Paracrine co-delivery of TGF-beta and IL-2 using CD4-targeted nanoparticles for induction and maintenance of regulatory T cells. Biomaterials. (2015) 59:172–81. 10.1016/j.biomaterials.2015.04.003 25974747 PMC5997248

[40] Lewis JS Roche C Zhang Y Brusko TM Wasserfall CH Atkinson M. Combinatorial delivery of immunosuppressive factors to dendritic cells using dual-sized microspheres. J Mater Chem B Mater Biol Med. (2014) 2:2562–74. 10.1039/C3TB21460E 24778809 PMC4000038

[41] Abiru N Maniatis AK Yu L Miao D Moriyama H Wegmann D. Peptide and major histocompatibility complex-specific breaking of humoral tolerance to native insulin with the B9-23 peptide in diabetes-prone and normal mice. Diabetes. (2001) 50:1274–81. 10.2337/diabetes.50.6.1274 11375327

[42] Eckenrode SE Ruan QG Collins CD Yang P McIndoe RA Muir A. Molecular pathways altered by insulin b9-23 immunization. Ann N Y Acad Sci. (2004) 1037:175–85. 10.1196/annals.1337.029 15699514

[43] Harrison LC Solly NR Martinez NR. (Pro)insulin-specific regulatory T cells. Novartis Found Symp. (2003) 252:132–41; discussion 141−5, 203–10. 14609216

[44] Nakayama M Babaya N Miao D Gianani R Liu E Elliott JF. Long-term prevention of diabetes and marked suppression of insulin autoantibodies and insulitis in mice lacking native insulin B9-23 sequence. Ann N Y Acad Sci. (2006) 1079:122–9. 10.1196/annals.1375.018 17130542

[45] Jia L Kovacs JR Zheng Y Gawalt ES Shen H Meng WS. Attenuated alloreactivity of dendritic cells engineered with surface-modified microspheres carrying a plasmid encoding interleukin-10. Biomaterials. (2006) 27:2076–82. 10.1016/j.biomaterials.2005.09.032 16219347

[46] Jia L Kovacs JR Zheng Y Shen H Gawalt ES Meng WS. Expansion of Foxp3-expressing regulatory T cells *in vitro* by dendritic cells modified with polymeric particles carrying a plasmid encoding interleukin-10. Biomaterials. (2008) 29:1250–61. 10.1016/j.biomaterials.2007.11.015 18086497

[47] Kovacs JR Tidball J Ross A Jia L Zheng Y Gawalt ES. Characterization of nickel-decorated PLGA particles anchored with a his-tagged polycation. J Biomater Sci Polym Ed. (2009) 20:1307–20. 10.1163/156856209X453015 19520014

[48] Zheng Y Kovacs JR Gawalt ES Shen H Meng WS. Characterization of particles fabricated with poly(D, L-lactic-co-glycolic acid) and an ornithine-histidine peptide as carriers of oligodeoxynucleotide for delivery into primary dendritic cells. J Biomater Sci Polym Ed. (2006) 17:1389–403. 10.1163/156856206778937217 17260510

[49] Zheng Y Wen Y George AM Steinbach AM Phillips BE Giannoukakis N. A peptide-based material platform for displaying antibodies to engage T cells. Biomaterials. (2011) 32:249–57. 10.1016/j.biomaterials.2010.08.083 20880580

[50] Broggi A Cigni C Zanoni I Granucci F. Preparation of single-cell suspensions for cytofluorimetric analysis from different mouse skin regions. J Vis Exp. (2016) 110:e52589. 10.3791/52589 27166881 PMC4941935

[51] Bouaziz JD Yanaba K Tedder TF. Regulatory B cells as inhibitors of immune responses and inflammation. Immunol Rev. (2008) 224:201–14. 10.1111/j.1600-065X.2008.00661.x 18759928

[52] Hong C Gao XM. Purification and immunophenotypic characterization of murine B10 B cells. Methods Mol Biol. (2014) 1190:35–44. 10.1007/978-1-4939-1161-5_3 25015271

[53] Karim MR Wang YF. Phenotypic identification of CD19(+)CD5(+)CD1d(+) regulatory B cells that produce interleukin 10 and transforming growth factor beta1 in human peripheral blood. Arch Med Sci. (2019) 15:1176–83. 10.5114/aoms.2018.77772 31572462 PMC6764295

[54] Matsushita T Tedder TF. Identifying regulatory B cells (B10 cells) that produce IL-10 in mice. Methods Mol Biol. (2011) 677:99–111. 10.1007/978-1-60761-869-0_7 20941605

[55] Yanaba K Bouaziz JD Haas KM Poe JC Fujimoto M Tedder TF. A regulatory B cell subset with a unique CD1dhiCD5+ phenotype controls T cell-dependent inflammatory responses. Immunity. (2008) 28:639–50. 10.1016/j.immuni.2008.03.017 18482568

[56] Yanaba K Bouaziz JD Matsushita T Tsubata T Tedder TF. The development and function of regulatory B cells expressing IL-10 (B10 cells) requires antigen receptor diversity and TLR signals. J Immunol. (2009) 182:7459–72. 10.4049/jimmunol.0900270 19494269 PMC3733128

[57] Tsang ML Zhou L Zheng BL Wenker J Fransen G Humphrey J. Characterization of recombinant soluble human transforming growth factor-beta receptor type II (rhTGF-beta sRII). Cytokine. (1995) 7:389–97. 10.1006/cyto.1995.0054 7578976

[58] Lewis JS Dolgova NV Zhang Y Xia CQ Wasserfall CH Atkinson MA. A combination dual-sized microparticle system modulates dendritic cells and prevents type 1 diabetes in prediabetic NOD mice. Clin Immunol. (2015) 160:90–102. 10.1016/j.clim.2015.03.023 25842187 PMC4554803

[59] Lewis JS Stewart JM Marshall GP Carstens MR Zhang Y Dolgova NV. Dual-sized microparticle system for generating suppressive dendritic cells prevents and reverses type 1 diabetes in the nonobese diabetic mouse model. ACS Biomater Sci Eng. (2019) 5:2631–46. 10.1021/acsbiomaterials.9b00332 31119191 PMC6518351

[60] Suwandi JS Toes RE Nikolic T Roep BO. Inducing tissue specific tolerance in autoimmune disease with tolerogenic dendritic cells. Clin Exp Rheumatol. (2015) 33(4 Suppl. 92):S97–103. 26458178

[61] Creusot RJ Chang P Healey DG Tcherepanova IY Nicolette CA Fathman CG. A short pulse of IL-4 delivered by DCs electroporated with modified mRNA can both prevent and treat autoimmune diabetes in NOD mice. Mol Ther. (2010) 18:2112–20. 10.1038/mt.2010.146 20628358 PMC2997578

[62] Yoon YM Lewis JS Carstens MR Campbell-Thompson M Wasserfall CH Atkinson MA. A combination hydrogel microparticle-based vaccine prevents type 1 diabetes in non-obese diabetic mice. Sci Rep. (2015) 5:13155. 10.1038/srep13155 26279095 PMC4538389

[63] Keselowsky BG Xia CQ Clare-Salzler M. Multifunctional dendritic cell-targeting polymeric microparticles: engineering new vaccines for type 1 diabetes. Hum Vaccin. (2011) 7:37–44. 10.4161/hv.7.1.12916 21157186 PMC3679212

[64] Battaglia M Ahmed S Anderson MS Atkinson MA Becker D Bingley PJ. Introducing the endotype concept to address the challenge of disease heterogeneity in type 1 diabetes. Diabetes Care. (2019) 43:5–12. 10.2337/dc19-0880 31753960 PMC6925574

[65] Battaglia M Atkinson MA. The streetlight effect in type 1 diabetes. Diabetes. (2015) 64:1081–90. 10.2337/db14-1208 25805758 PMC4375074

[66] Battaglia M Petrelli A Vecchio F. Neutrophils and type 1 diabetes: current knowledge and suggested future directions. Curr Opin Endocrinol Diabetes Obes. (2019) 26:201–6. 10.1097/MED.0000000000000485 31157631

[67] Campbell-Thompson M Rodriguez-Calvo T Battaglia M. Abnormalities of the exocrine pancreas in type 1 diabetes. Curr Diab Rep. (2015) 15:79. 10.1007/s11892-015-0653-y 26318606 PMC5072278

[68] Valle A Giamporcaro GM Scavini M Stabilini A Grogan P Bianconi E. Reduction of circulating neutrophils precedes and accompanies type 1 diabetes. Diabetes. (2013) 62:2072–7. 10.2337/db12-1345 23349491 PMC3661622

[69] Vecchio F Lo Buono N Stabilini A Nigi L Dufort MJ Geyer S. Type 1 diabetes trialnet study, Battaglia M. Abnormal neutrophil signature in the blood and pancreas of presymptomatic and symptomatic type 1 diabetes. JCI Insight. (2018) 3. 10.1172/jci.insight.122146 30232284 PMC6237216

[70] Kolb H von Herrath M. Immunotherapy for type 1 diabetes: why do current protocols not halt the underlying disease process? Cell Metab. (2017) 25:233–41. 10.1016/j.cmet.2016.10.009 27839907

[71] Kroger CJ Clark M Ke Q Tisch RM. Therapies to suppress beta cell autoimmunity in type 1 diabetes. Front Immunol. (2018) 9:1891. 10.3389/fimmu.2018.01891 30166987 PMC6105696

[72] Roep BO. Are insights gained from NOD mice sufficient to guide clinical translation? Another inconvenient truth. Ann N Y Acad Sci. (2007) 1103:1–10. 10.1196/annals.1394.018 17376838

[73] Couri CE Oliveira MC Stracieri AB Moraes DA Pieroni F Barros GM. C-peptide levels and insulin independence following autologous nonmyeloablative hematopoietic stem cell transplantation in newly diagnosed type 1 diabetes mellitus. JAMA. (2009) 301:1573–9. 10.1001/jama.2009.470 19366777

[74] Haller MJ Gitelman SE Gottlieb PA Michels AW Perry DJ Schultz AR. Antithymocyte globulin plus G-CSF combination therapy leads to sustained immunomodulatory and metabolic effects in a subset of responders with established type 1 diabetes. Diabetes. (2016) 65:3765–75. 10.2337/db16-0823 27669730 PMC5127248

[75] Haller MJ Gitelman SE Gottlieb PA Michels AW Rosenthal SM Shuster JJ. Anti-thymocyte globulin/G-CSF treatment preserves beta cell function in patients with established type 1 diabetes. J Clin Invest. (2015) 125:448–55. 10.1172/JCI78492 25500887 PMC4382237

[76] Herold KC Hagopian W Auger JA Poumian-Ruiz E Taylor L Donaldson D. Anti-CD3 monoclonal antibody in new-onset type 1 diabetes mellitus. N Engl J Med. (2002) 346:1692–8. 10.1056/NEJMoa012864 12037148

[77] Pescovitz MD Greenbaum CJ Krause-Steinrauf H Becker DJ Gitelman SE Goland R. Type 1 diabetes trialnet anti: rituximab, B-lymphocyte depletion, and preservation of beta-cell function. N Engl J Med. (2009) 361:2143–52. 10.1056/NEJMoa0904452 19940299 PMC6410357

[78] Ryan EA Paty BW Senior PA Bigam D Alfadhli E Kneteman NM. Five-year follow-up after clinical islet transplantation. Diabetes. (2005) 54:2060–9. 10.2337/diabetes.54.7.2060 15983207

[79] Sherry N Hagopian W Ludvigsson J Jain SM Wahlen J Ferry RJ. Protege Trial, Teplizumab for treatment of type 1 diabetes (Protege study): 1-year results from a randomised, placebo-controlled trial. Lancet. (2011) 378:487–97. 10.1016/S0140-6736(11)60931-8 21719095 PMC3191495

[80] Voltarelli JC Couri CE Stracieri AB Oliveira MC Moraes DA Pieroni F. Autologous nonmyeloablative hematopoietic stem cell transplantation in newly diagnosed type 1 diabetes mellitus. JAMA. (2007) 297:1568–76. 10.1001/jama.297.14.1568 17426276

[81] Casey LM Pearson RM Hughes KR Liu JMH Rose JA North MG. Conjugation of transforming growth factor beta to antigen-loaded poly(lactide- co-glycolide) nanoparticles enhances efficiency of antigen-specific tolerance. Bioconjug Chem. (2018) 29:813–23. 10.1021/acs.bioconjchem.7b00624 29148731 PMC6850215

[82] Cho JJ Stewart JM Drashansky TT Brusko MA Zuniga AN Lorentsen KJ. An antigen-specific semi-therapeutic treatment with local delivery of tolerogenic factors through a dual-sized microparticle system blocks experimental autoimmune encephalomyelitis. Biomaterials. (2017) 143:79–92. 10.1016/j.biomaterials.2017.07.029 28772190 PMC5870833

[83] Getts DR Martin AJ McCarthy DP Terry RL Hunter ZN Yap WT. Microparticles bearing encephalitogenic peptides induce T-cell tolerance and ameliorate experimental autoimmune encephalomyelitis. Nat Biotechnol. (2012) 30:1217–24. 10.1038/nbt.2434 23159881 PMC3589822

[84] Hlavaty KA Luo X Shea LD Miller SD. Cellular and molecular targeting for nanotherapeutics in transplantation tolerance. Clin Immunol. (2015) 160:14–23. 10.1016/j.clim.2015.03.013 25805659 PMC4554810

[85] Hunter Z McCarthy DP Yap WT Harp CT Getts DR Shea LD. A biodegradable nanoparticle platform for the induction of antigen-specific immune tolerance for treatment of autoimmune disease. ACS Nano. (2014) 8:2148–60. 10.1021/nn405033r 24559284 PMC3990004

[86] Kuo R Saito E Miller SD Shea LD. Peptide-conjugated nanoparticles reduce positive co-stimulatory expression and T cell activity to induce tolerance. Mol Ther. (2017) 25:1676–85. 10.1016/j.ymthe.2017.03.032 28408181 PMC5498812

[87] Luo X Miller SD Shea LD. Immune tolerance for autoimmune disease and cell transplantation. Annu Rev Biomed Eng. (2016) 18:181–205. 10.1146/annurev-bioeng-110315-020137 26928211 PMC4947009

[88] McCarthy DP Yap JW Harp CT Song WK Chen J Pearson RM. An antigen-encapsulating nanoparticle platform for TH1/17 immune tolerance therapy. Nanomedicine. (2017) 13:191–200. 10.1016/j.nano.2016.09.007 27720992 PMC5237397

[89] Pearson RM Casey LM Hughes KR Miller SD Shea LD. In vivo reprogramming of immune cells: technologies for induction of antigen-specific tolerance. Adv Drug Deliv Rev. (2017) 114:240–55. 10.1016/j.addr.2017.04.005 28414079 PMC5582017

[90] Mukherjee G Geliebter A Babad J Santamaria P Serreze DV Freeman GJ. DEC-205-mediated antigen targeting to steady-state dendritic cells induces deletion of diabetogenic CD8(+) T cells independently of PD-1 and PD-L1. Int Immunol. (2013) 25:651–60. 10.1093/intimm/dxt031 24021877 PMC3806169

[91] Mukhopadhaya A Hanafusa T Jarchum I Chen YG Iwai Y Serreze DV. Selective delivery of beta cell antigen to dendritic cells in vivo leads to deletion and tolerance of autoreactive CD8+ T cells in NOD mice. Proc Natl Acad Sci USA. (2008) 105:6374–9. 10.1073/pnas.0802644105 18430797 PMC2359791

[92] Jamison BL Neef T Goodspeed A Bradley B Baker RL Miller SD. Nanoparticles containing an insulin-ChgA hybrid peptide protect from transfer of autoimmune diabetes by shifting the balance between effector T cells and regulatory T cells. J Immunol. (2019) 203:48–57. 10.4049/jimmunol.1900127 31109955 PMC6581587

[93] Bai A Lu N Guo Y Liu Z Chen J Peng Z. All-trans retinoic acid down-regulates inflammatory responses by shifting the Treg/Th17 profile in human ulcerative and murine colitis. J Leukoc Biol. (2009) 86:959–69. 10.1189/jlb.0109006 19477911

[94] Benson MJ Pino-Lagos K Rosemblatt M Noelle RJ. All-trans retinoic acid mediates enhanced T reg cell growth, differentiation, and gut homing in the face of high levels of co-stimulation. J Exp Med. (2007) 204:1765–74. 10.1084/jem.20070719 17620363 PMC2118687

[95] Hall JA Grainger JR Spencer SP Belkaid Y. The role of retinoic acid in tolerance and immunity. Immunity. (2011) 35:13–22. 10.1016/j.immuni.2011.07.002 21777796 PMC3418663

[96] Iwata M Yokota A. Retinoic acid production by intestinal dendritic cells. Vitam Horm. (2011) 86:127–52. 10.1016/B978-0-12-386960-9.00006-X 21419270

[97] Lu L Ma J Li Z Lan Q Chen M Liu Y. All-trans retinoic acid promotes TGF-beta-induced Tregs via histone modification but not DNA demethylation on Foxp3 gene locus. PLoS ONE. (2011) 6:e24590. 10.1371/journal.pone.0024590 21931768 PMC3172235

[98] Mucida D Park Y Kim G Turovskaya O Scott I Kronenberg M. Reciprocal TH17 and regulatory T cell differentiation mediated by retinoic acid. Science. (2007) 317:256–60. 10.1126/science.1145697 17569825

[99] Sakaguchi S Miyara M Costantino CM Hafler DA. FOXP3+ regulatory T cells in the human immune system. Nat Rev Immunol. (2010) 10:490–500. 10.1038/nri2785 20559327

[100] Wing K Sakaguchi S. Regulatory T cells exert checks and balances on self tolerance and autoimmunity. Nat Immunol. (2010) 11:7–13. 10.1038/ni.1818 20016504

[101] Jordan MS Boesteanu A Reed AJ Petrone AL Holenbeck AE Lerman MA. Thymic selection of CD4+CD25+ regulatory T cells induced by an agonist self-peptide. Nat Immunol. (2001) 2:301–6. 10.1038/86302 11276200

[102] Kawahata K Misaki Y Yamauchi M Tsunekawa S Setoguchi K Miyazaki J. Generation of CD4(+)CD25(+) regulatory T cells from autoreactive T cells simultaneously with their negative selection in the thymus and from nonautoreactive T cells by endogenous TCR expression. J Immunol. (2002) 168:4399–405. 10.4049/jimmunol.168.9.4399 11970982

[103] Curotto de Lafaille MA Lafaille JJ. Natural and adaptive foxp3+ regulatory T cells: more of the same or a division of labor? Immunity. (2009) 30:626–35. 10.1016/j.immuni.2009.05.002 19464985

[104] Coombes JL Maloy KJ. Control of intestinal homeostasis by regulatory T cells and dendritic cells. Semin Immunol. (2007) 19:116–26. 10.1016/j.smim.2007.01.001 17320411

[105] Sun CM Hall JA Blank RB Bouladoux N Oukka M Mora JR. Small intestine lamina propria dendritic cells promote de novo generation of Foxp3 T reg cells via retinoic acid. J Exp Med. (2007) 204:1775–85. 10.1084/jem.20070602 17620362 PMC2118682

[106] Koprivica I Gajic D Saksida T Cavalli E Auci D Despotovic S. Orally delivered all-trans-retinoic acid- and transforming growth factor-beta-loaded microparticles ameliorate type 1 diabetes in mice. Eur J Pharmacol. (2019) 864:172721. 10.1016/j.ejphar.2019.172721 31586630

[107] Atkinson MA Leiter EH. The NOD mouse model of type 1 diabetes: as good as it gets? Nat Med. (1999) 5:601–4. 10.1038/9442 10371488

[108] Chen YG Mathews CE Driver JP. The role of NOD mice in type 1 diabetes research: lessons from the past and recommendations for the future. Front Endocrinol. (2018) 9:51. 10.3389/fendo.2018.00051 29527189 PMC5829040

[109] Pearson JA Wong FS Wen L. The importance of the Non Obese Diabetic (NOD) mouse model in autoimmune diabetes. J Autoimmun. (2016) 66:76–88. 10.1016/j.jaut.2015.08.019 26403950 PMC4765310

[110] Reed JC Herold KC. Thinking bedside at the bench: the NOD mouse model of T1DM. Nat Rev Endocrinol. (2015) 11:308–14. 10.1038/nrendo.2014.236 25623120 PMC4523382

