# Supplementary figures and images for: Arrest in the Progression of Type 1 Diabetes at the Mid-Stage of Insulitic Autoimmunity Using an Autoantigen-Decorated All-trans Retinoic Acid and Transforming Growth Factor Beta-1 Single Microparticle Formulation
Source: Front Immunol. 2021 Mar 8;12:586220. doi: 10.3389/fimmu.2021.586220 (PMC7982719; doi:10.3389/fimmu.2021.586220)

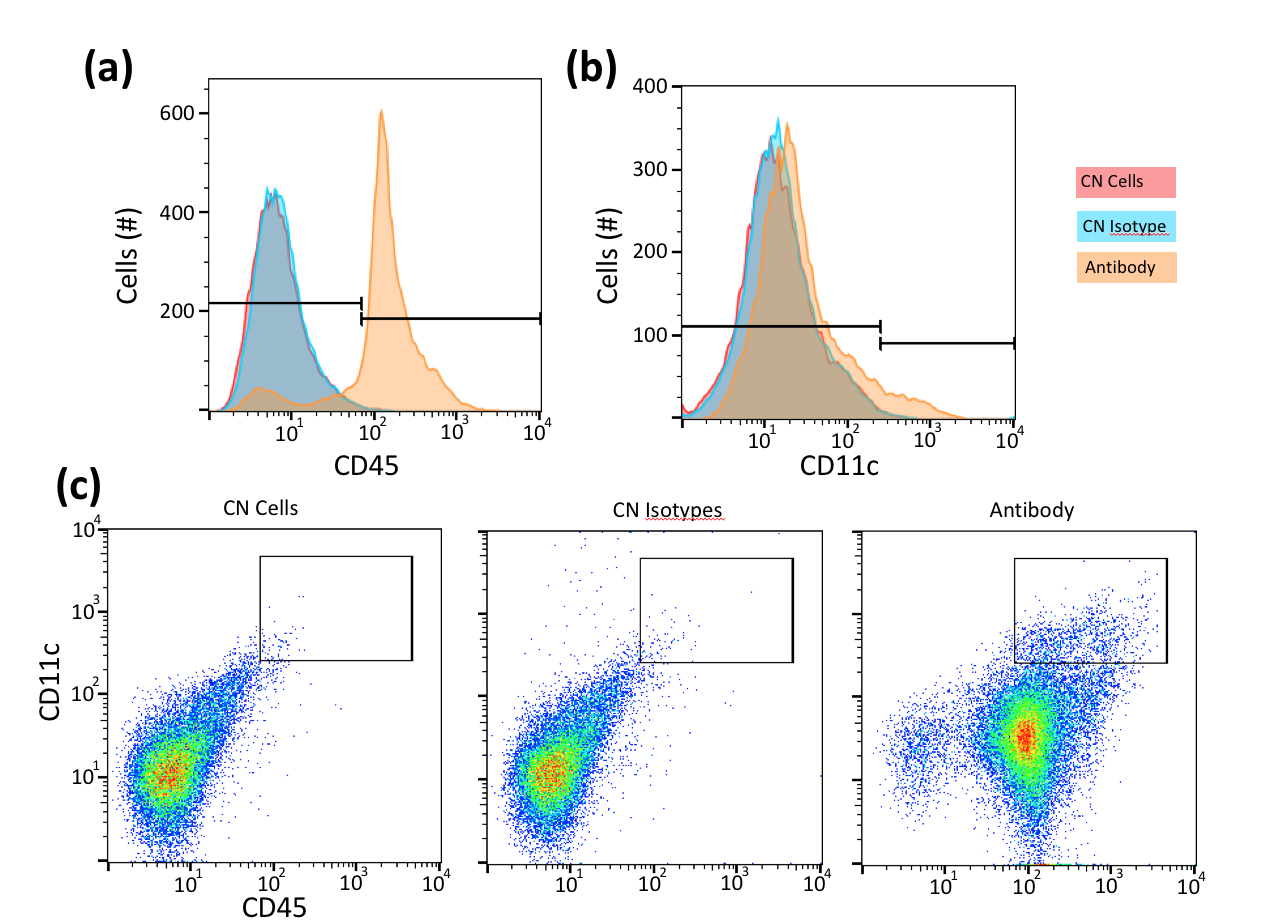

Supplement: Supplementary Figure 1 — Dendritic cell antibody isotypes and controls used in flow cytometry. Single cell suspensions of splenocytes were isolated and grown in vitro. Splenocytes were analyzed by flow cytometry to identify dendritic cells using the CD45 and CD11c cell surface markers 18 h later. Fluorescence histograms of unstained cells are shown in red and cells stained with matched fluorochrome-conjugated isotypes are shown in blue. Cells with antibody against (A) CD45 or (B) CD11c are shown in orange. A signal intensity cut off is shown which displays positively stained cells to the right. (C) Signal intensity is shown in both CD45 and CD11c channels, with CD45+ CD11c+ cells located inside the box. [file Image_1.TIF]

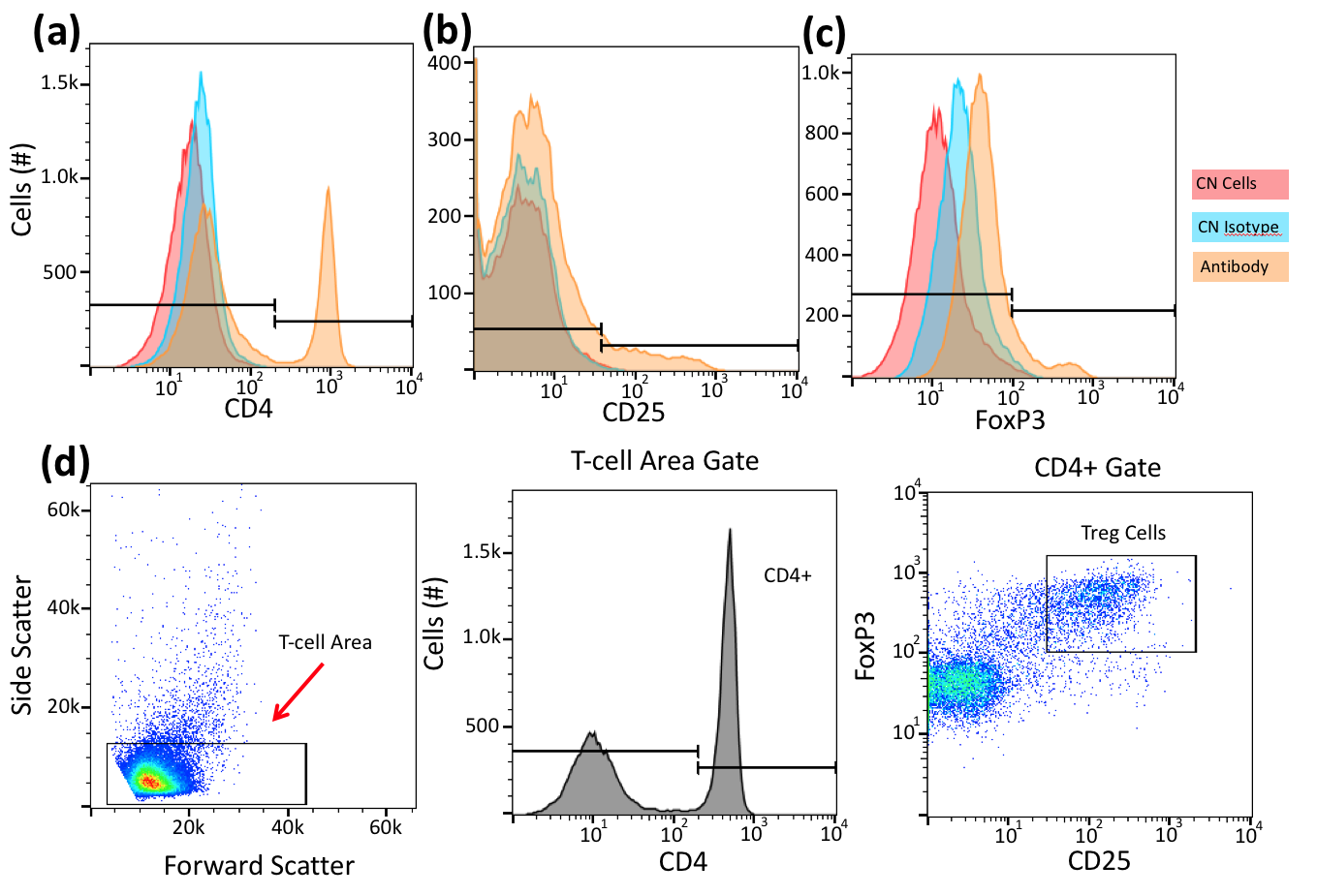

Supplement: Supplementary Figure 2 — Antibody controls and gating method in Treg flow cytometry. Single cell suspensions of lymph nodes were analyzed by flow cytometry to identify Tregs by cell surface markers CD4, CD25, and nuclear marker FoxP3. Histograms of control unstained cells are shown in red and cells stained with matched fluorochrome-conjugated isotypes are shown in blue. Cells with antibody against (A) CD4, (B) CD25, (C) FoxP3 are shown in orange. A signal intensity cut off is shown which displays positively stained cells to the right. (D) The gating method for identifying Foxp3+ Tregs is shown. First, cells are selected for low granularity based on low side scatter. Within this population, CD4+ cells are selected as a general T-cell population. Finally, within the CD4+ cell population, cells that are CD25+ FoxP3+ are selected for further analysis and quantitation. [file Image_2.TIF]

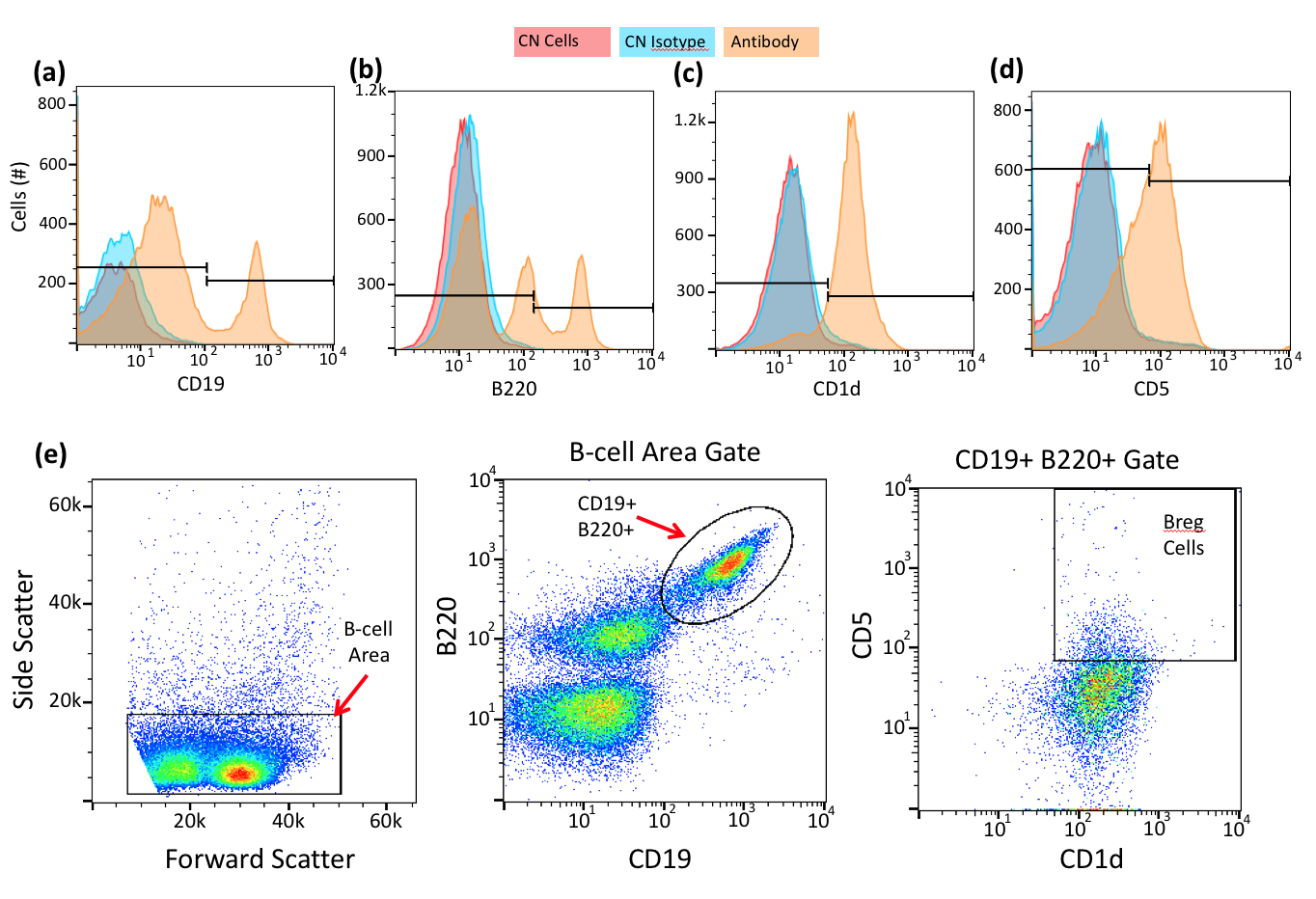

Supplement: Supplementary Figure 3 — Breg antibody controls and gating method in Breg flow cytometry. Single cell suspensions of lymph nodes were analyzed by flow cytometry to identify Bregs by cell surface markers CD19, B220, CD1d, and CD5. Histograms of control unstained cells are shown in red and cells stained with matched fluorochrome-conjugated isotypes are shown in blue. Cells with antibody against (A) CD19, (B) B220, (C) CD1d, and (D) CD5 are shown in orange. A signal intensity cut off is shown which displays positively stained cells to the right. (E) The gating method for identifying Bregs is shown. First, cells are selected for low granularity as indicated by low side scatter. Within this population, the prevalent CD19+ B220+ double positive B-cells are selected. Finally, within this B cell population, cells that are CD1d+ CD5+ are selected as shown for further analysis. [file Image_3.TIF]

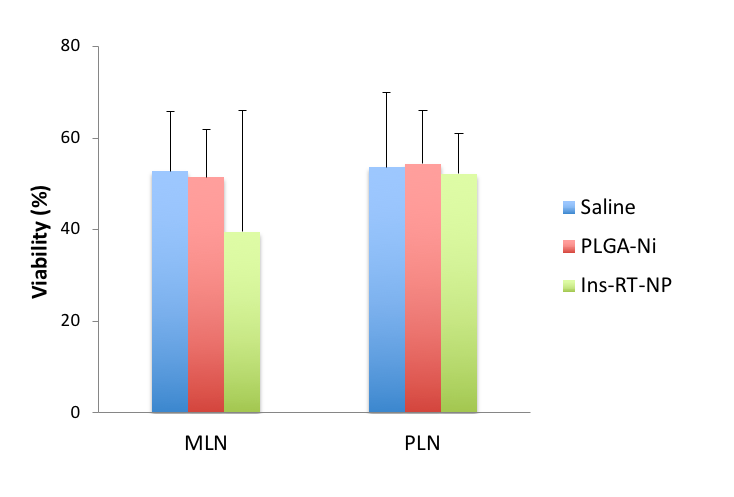

Supplement: Supplementary Figure 4 — Lymph node (LN) cell viability. Animals received a single injection of saline, 10 mg PLGA-Ni, or 10 mg Ins-RT-NP. Single cell suspensions were made from MLN and PLN. Cell viability was assessed after LN dissociation by Trypan Blue staining. The percentage of viable cells is displayed and no significant differences in viability were detected (individual one-way ANOVA per LN type). Experiments include two separate animal cohorts combined (n = 9). [file Image_4.TIF]
